# Clinical features and recurrence risk prediction model in patients with idiopathic inflammatory myopathies-associated interstitial lung disease: a retrospective study from Ningbo

**DOI:** 10.3389/fmed.2025.1666332

**Published:** 2025-09-10

**Authors:** Di Wu, Yapeng Hou, Wei Fan, Yingying Du, Qunli Ding

**Affiliations:** ^1^Key Laboratory of Respiratory Disease of Ningbo, Department of Respiratory and Critical Care Medicine, The First Affiliated Hospital of Ningbo University, Ningbo, China; ^2^Medical College of Ningbo University, Ningbo, Zhejiang, China

**Keywords:** IIM-ILD, recurrence, risk prediction model, AUC, Ningbo

## Abstract

**Background:**

Idiopathic inflammatory myopathies (IIMs) are characterized by chronic muscle inflammation and often involve multiple organ systems. Despite treatment, a large number of patients still experience relapse or disease progression. Currently, there is a lack of high-quality studies specifically focusing on recurrence, and early identification of patients at high risk of recurrence remains challenging.

**Methods:**

This retrospective study included patients with idiopathic inflammatory myopathy complicated by interstitial lung disease who were hospitalized in the First Affiliated Hospital of Ningbo University from January 2018 to December 2024. All included individuals were followed up for at least 12 months. The diagnosis of idiopathic inflammatory myopathies and interstitial lung disease (ILD) was based on relevant criteria. A wide range of clinical and laboratory data were collected. Significant variables were screened through univariate and multivariate regression analyses, and risk models for predicting recurrence at 1, 2, and 3 years were constructed, with subsequent evaluation of the models’ performance.

**Results:**

Among the 93 included patients, the recurrence rate was 23.7%, with a median time to recurrence of 38 months. Approximately 26.1% of recurrences were attributed to drug discontinuation or adjustment. Multivariate analysis suggested that positive anti-RO52 antibody, positive anti-PL7 antibody, elevated white blood cell count, elevated ALT, and elevated LDH were positively correlated with the risk of recurrence. The area under the ROC curve (AUC) for recurrence prediction was: 1-year: 1.00 (95% CI: 1.00–1.00), 2-year: 0.91 (95% CI: 0.75–1.08), 3-year: 0.92 (95% CI: 0.78–1.05).

**Conclusion:**

Based on baseline clinical and laboratory indicators, this study developed a tool with good predictive ability for IIM-ILD recurrence, which can accurately assess individual recurrence risk and assist in early clinical decision-making.

**Trial registration number:**

IRB No:2025092RS.

## Introduction

Idiopathic inflammatory myopathies (IIMs) represent a diverse category of autoimmune disorders. These conditions are defined by persistent muscle inflammation. Common symptoms include symmetrical proximal muscle weakness, muscle soreness, and muscle atrophy (1). IIMs primarily include polymyositis, dermatomyositis, and inclusion body myositis (2). Anti-synthetase syndrome is also a specific subtype of idiopathic inflammatory myopathies (IIMs), and it is an autoimmune disease associated with anti-aminoacyl-tRNA synthetase antibodies. In addition to muscle involvement, IIM often affects multiple systems, including the skin, lungs, and kidneys. Clinical features may include fever, Raynaud’s phenomenon, Gottron’s sign, V-sign, and shawl sign (3). Interstitial lung disease (ILD) is a hallmark of pulmonary involvement, and ILD precedes muscle symptoms in up to 20% of cases (4).

ILD is the main cause of death and hospitalization in patients with inflammatory myopathies, and patients with IIMs complicated by ILD have a significantly higher risk of death or acute disease exacerbation (5–7). The most common interstitial lung disease (ILD) patterns associated with idiopathic inflammatory myopathies (IIMs) are nonspecific interstitial pneumonia (NSIP) and organizing pneumonia (OP). NSIP can be divided into cellular and fibrotic types, both characterized by symmetric ground-glass opacities in the bilateral lung bases, with the fibrotic type also accompanied by fine reticular structures. OP is characterized by ground-glass consolidation, rarely showing reticular opacities, bronchiectasis, etc. The lesions are mostly distributed bilaterally in a subpleural/perihilar and perisegmental pattern, especially in the mid-basal lung regions. In addition, the usual interstitial pneumonia (UIP) pattern is uncommon in IIM-ILD, featuring traction bronchiectasis, honeycombing, and thickening of bronchovascular bundles (8). Thoracic complications, including tracheoesophageal fistula and pneumothorax, may further increase the mortality risk in IIM-ILD patients (9–12).

Current treatment strategies for IIM-ILD include glucocorticoids and immunosuppressive agents such as methotrexate, cyclophosphamide, tacrolimus, and rituximab (13–16). Although combination therapy can improve acute conditions in most patients, 20–50% still experience recurrence or disease progression during treatment, characterized by worsening dyspnea, progressive lung function decline, and even respiratory failure. Such recurrence significantly increases disability and mortality rates, posing challenges to clinical management (17, 18). Studies have confirmed that reduced vital capacity and anti-aminoacyl-tRNA synthetase (anti-ARS) antibodies are risk factors for recurrence in patients (19). Meanwhile, patients’ partial response to treatment is also a risk factor for poor prognosis and disease progression (20).

However, high-quality studies on recurrence risk in IIM-ILD are still lacking, and early identification of high-risk patients remains difficult. Accurate prognostic risk assessment is essential for clinical decision-making. While several models have been developed to predict adverse outcomes such as death in IIM-ILD, none have specifically addressed recurrence prediction (21–23). This study aims to construct a clinical prediction model for the recurrence of IIM-ILD using laboratory test data, pulmonary function data, and imaging data of IIM-ILD patients, identify high-risk factors for disease recurrence, and provide support for the management of IIM-ILD.

## Methods

### Patient selection

We conducted a retrospective cohort study on patients with IIM-associated interstitial lung disease who were hospitalized in the First Affiliated Hospital of Ningbo University from January 2018 to December 2024. The diagnosis of ILD was based on clinical symptoms and characteristic imaging findings. In the case of autoimmune diseases such as systemic sclerosis, the interstitial lung disease (ILD) they are complicated with differs significantly from isolated IIM-ILD in terms of pathogenesis, clinical course, treatment modalities, and prognostic features. These diseases themselves have unique risk factors for ILD progression, which are quite distinct from the recurrence-related factors of IIM-ILD. Therefore, we will not include these populations. The exclusion criteria were as follows:

1. Presence of comorbidities such as heart failure, respiratory failure, or malignancies;
2. Coexisting connective tissue diseases, including rheumatoid arthritis, systemic sclerosis, and Sjögren’s syndrome;
3. Missing critical clinical data, such as high-resolution CT scans;
4. ILD is attributed to secondary causes such as drug-induced toxicity or infection.

**Figure 1:**
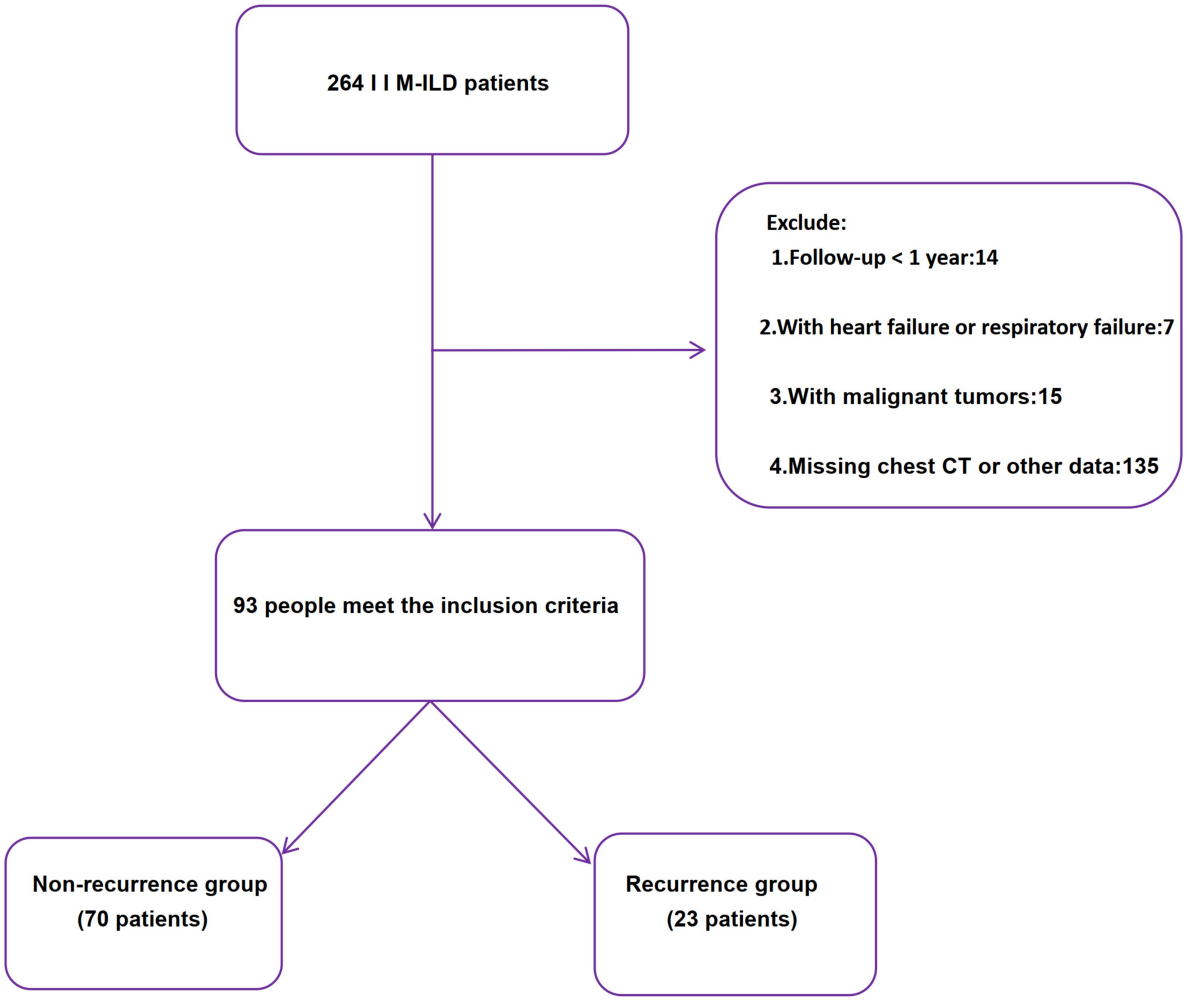
Flow chart for study selection.

All patients were followed for a minimum of 12 months. This study was approved by the Ethics Committee of the First Affiliated Hospital of Ningbo University. Due to the retrospective nature and anonymized data, informed consent was waived (Figure 1).

### Diagnostic criteria for IIM-ILD and recurrence

The diagnosis of PM/DM is established based on the 1975 Bohan and Peter diagnostic criteria and the 2017 EULAR/ACR classification criteria (23, 24). Anti-synthetase syndrome is defined as the presence of anti-aminoacyl-tRNA synthetase antibodies, accompanied by one or more of the following symptoms: 1. Interstitial lung disease (ILD); 2. Fever; 3. Arthritis; 4. Muscle involvement symptoms such as symmetric proximal muscle weakness, muscle pain or tenderness; 5. Mechanic’s hands (25). The diagnosis of interstitial lung disease (ILD) was based on a combination of clinical and diagnostic findings, including respiratory symptoms, physical examination (presence of Velcro crackles), and characteristic abnormalities on high-resolution computed tomography (HRCT), such as consolidation, reticular opacities, and honeycombing. Pulmonary function tests demonstrated restrictive ventilatory impairment and reduced gas exchange capacity, defined as total lung capacity (TLC) and diffusing capacity for carbon monoxide (DLCO) both below 80% of the predicted values (26). Recurrence of IIM-ILD was defined as progressive ILD requiring an escalation of glucocorticoid dosage during treatment. Disease progression is defined according to the criteria for progressive pulmonary fibrosis (PPF), requiring the presence of at least two of the following three features within the preceding year: 1. Worsening of respiratory symptoms or extrapulmonary symptoms; 2. Physiological evidence of disease progression, indicated by: a. An absolute decline in forced vital capacity (FVC) of ≥5% of predicted value; b. An absolute decline in DLCO of ≥10% of predicted value; 3. Radiological evidence of progression on HRCT, including one or more of the following: a. Increased extent or severity of traction bronchiectasis and lobular dilatation; b. Newly developed ground-glass opacities with traction bronchiectasis; c. Newly appeared fine reticular opacities. Increased extent or thickness of reticular abnormalities; e. Newly appeared or increased honeycombing; f. Progressive lobar volume loss (27, 28). The HRCT scans of patients were independently evaluated by two expert radiologists in the study.

### Data extraction

The clinical data were extracted from the original medical records before the initiation of treatment. Upon hospital admission, all patients underwent immunoblot testing in the hospital laboratory to detect relevant autoantibodies. Test results marked as “+++” or “++” were classified as positive, while all other results were considered negative. Collected demographic and clinical information including age, sex, disease duration, and the presence of symptoms such as productive cough and arthralgia. Laboratory assessments included neutrophil count, red blood cell count, white blood cell count, lactate dehydrogenase (LDH), complement C3, C-reactive protein (CRP), immunoglobulin G (IgG), immunoglobulin A (IgA), immunoglobulin M (IgM), erythrocyte sedimentation rate (ESR), and creatine kinase (CK). Arterial blood gas analysis (PaO₂, PaCO₂, pH) and pulmonary function test results, including forced expiratory volume in one second (FEV1%), forced vital capacity (FVC%), and diffusing capacity for carbon monoxide (DLCO%), were also collected. All patients underwent chest high-resolution computed tomography (HRCT) at admission. Radiographic patterns were categorized as nonspecific interstitial pneumonia (NSIP), organizing pneumonia (OP), or usual interstitial pneumonia (UIP). Additionally, medication records were reviewed to determine the use of immunosuppressive agents such as tacrolimus, cyclophosphamide, and cyclosporine. Glucocorticoid dosages were also recorded and standardized by converting all regimens to prednisone equivalents for uniform comparison.

### Statistical analysis

Continuous variables following a normal distribution were summarized as mean ± standard deviation (SD) and compared using independent t-tests. Non-normally distributed data were presented as interquartile ranges (IQR) and analyzed with the Mann–Whitney U test. Categorical variables were expressed as counts and percentages, and comparisons were made using the chi-square test or Fisher’s exact test, depending on applicability. For variables with low levels of missing data, multiple imputation was employed to minimize bias. To evaluate the body’s inflammatory indices, we introduced four composite indices: the neutrophil-to-lymphocyte ratio (NLR), the monocyte-to-lymphocyte ratio (MLR), the systemic immune-inflammation index (SII), and the systemic inflammation response index (SIRI). Based on the temporal distribution of recurrence events, we developed three predictive risk models for recurrence at 1, 2, and 3 years. Univariate logistic regression analyses were performed on patient demographics, clinical symptoms, laboratory test results, pulmonary function parameters, and HRCT imaging features. Variables with a *p* value < 0.1 were included in a stepwise multivariable logistic regression model to construct the final predictive models. The predictive performance of the resulting nomograms was evaluated in terms of discrimination, calibration, and clinical utility. Discrimination was assessed using receiver operating characteristic (ROC) curves, and the area under the curve (AUC) was used as the measure of predictive accuracy. To obtain robust estimates, bootstrapping with 1,000 resamples was performed to calculate the AUC and its corresponding 95% confidence interval (CI). Model calibration was evaluated using the Hosmer–Lemeshow goodness-of-fit test, Brier score, and calibration curves comparing predicted versus actual probabilities. Clinical applicability was examined through decision curve analysis (DCA). Based on the median nomogram score, patients were categorized into high and low-risk groups. To test the stability of the model, we excluded patients with missing original pulmonary function data, those with a short follow-up period (relative to the median time to recurrence), and those who modified their treatment regimens on their own initiative. We then re-established the prediction model and compared the diagnostic performance between the new model and the original one. Kaplan–Meier analysis was used to assess time-to-event outcomes and subgroup analyses were conducted according to key antibody status and risk stratification to explore differences in recurrence-free survival. All statistical analyses were conducted using R software (version 4.0.3). A two-tailed *p* value < 0.05 was considered statistically significant.

## Results

### Baseline characteristics

In terms of gender distribution, 20.43% (19/93) of the patients were male and 79.57% (74/93) were female. Laboratory analyses indicated that values for neutrophils, platelets, NLR, SII, SIRI, CK, AST, ALT, and LDH were generally higher in the recurrence group than in the non-recurrence group; however, none of these differences reached statistical significance. Pulmonary function parameters in the overall cohort showed a mean FEV1% predicted of 71.12 ± 22.31%, mean FVC% predicted of 71.52 ± 19.40%, and mean DLCO% predicted of 57.62 ± 35.09%. The recurrence group showed reduced FEV1% predicted and FVC% predicted values, whereas the non-recurrence group had a higher DLCO% predicted. It takes 4.23 months to reach the first stable phase after diagnosis. The recurrence group requires a longer time to achieve symptom stabilization (4.07 ± 2.34 months vs. 4.70 ± 1.96 months, *p* = 0.25).

However, none of these differences were statistically significant. Radiological findings revealed a significantly higher incidence of reticular shadows in the recurrence group compared to the non-recurrence group (86.96% vs. 38.57%, *Χ*^2^ = 5.16, *p* = 0.02). No significant differences were observed between groups in the prevalence of nodular shadows, honeycombing, ground-glass opacities, or in the distribution of interstitial lung disease (ILD) subtypes (all *p* > 0.05). Arterial blood gas analyses across the total population yielded a mean PaO₂ of 111.04 ± 36.35 mmHg, a mean PaCO₂ of 38.00 ± 4.40 mmHg, and a mean pH of 7.42 ± 0.05. No statistically significant differences were found between the recurrence and non-recurrence groups for any of these values (Table.1).

**Table 1:** Baseline clinical characteristics of IIM-ILD patients.

Variables	Total (*n* = 93)	Non-recurrence (*n* = 70)	Recurrence (*n* = 23)	Statistic	*P*
Sex, *n*(%)				*χ*^2^ = 0.51	0.47
Man	19 (20.43)	16 (22.86)	3 (13.04)		
Women	74 (79.57)	54 (77.14)	20 (86.96)		
Smoking, *n*(%)				*χ*^2^ = 0.51	0.47
NO	74 (79.57)	54 (77.14)	20 (86.96)		
YES	19 (20.43)	16 (22.86)	3 (13.04)		
Age, Mean ± SD(Years)	57.71 ± 12.63	57.13 ± 13.30	59.48 ± 10.38	*t* = −0.77	0.44
Leukocyte (× 109/L)	7.59 ± 3.74	7.54 ± 3.61	7.72 ± 4.21	*t* = −0.20	0.84
Monocyte (× 109/L)	0.59 ± 0.67	0.61 ± 0.75	0.54 ± 0.26	*t* = 0.45	0.66
Neutrophils (× 109/L)	5.80 ± 3.42	5.72 ± 3.34	6.04 ± 3.70	*t* = −0.40	0.69
Lymphocyte (× 109/L)	1.29 ± 0.74	1.32 ± 0.72	1.22 ± 0.83	*t* = 0.52	0.60
NLR	5.74 ± 5.77	5.56 ± 5.78	6.27 ± 5.83	*t* = −0.51	0.61
MLR	0.56 ± 0.94	0.58 ± 1.08	0.51 ± 0.19	*t* = 0.31	0.75
SIRI	3.17 ± 4.39	3.17 ± 4.79	3.19 ± 2.95	*t* = −0.02	0.99
SII	1559.74 ± 1915.57	1465.75 ± 1696.91	1845.78 ± 2489.97	*t* = −0.82	0.41
Erythrocyte (× 109/L)	4.32 ± 0.89	4.33 ± 0.97	4.27 ± 0.60	*t* = 0.29	0.77
Platelet (× 109/L)	255.45 ± 79.93	251.84 ± 78.73	266.43 ± 84.30	*t* = −0.76	0.45
ESR (mm/h)	33.68 ± 21.53	35.17 ± 23.01	29.13 ± 15.81	*t* = 1.17	0.25
CRP (mg/L)	5.86 ± 8.70	5.69 ± 8.31	6.40 ± 9.97	*t* = −0.34	0.74
Creatine kinase (U/L)	416.23 ± 1025.70	382.03 ± 816.77	520.30 ± 1514.00	*t* = −0.56	0.58
AST (U/L)	50.02 ± 57.78	45.29 ± 47.69	64.43 ± 80.86	*t* = −1.39	0.17
ALT (U/L)	39.83 ± 40.84	35.80 ± 33.76	52.09 ± 56.50	*t* = −1.68	0.10
LDH (U/L)	325.78 ± 211.82	314.50 ± 209.42	360.13 ± 220.08	*t* = −0.90	0.37
IgG (g/L)	13.53 ± 4.46	14.02 ± 4.56	12.04 ± 3.83	*t* = 1.87	0.06
IgM (g/L)	1.84 ± 1.91	1.91 ± 2.13	1.62 ± 0.94	*t* = 0.64	0.52
IgA (g/L)	2.74 ± 1.37	2.87 ± 1.41	2.33 ± 1.14	*t* = 1.66	0.10
C3 (g/L)	0.91 ± 0.26	0.91 ± 0.26	0.91 ± 0.27	*t* = 0.00	1.00
FEV1, %predicted	71.12 ± 22.31	71.42 ± 23.01	70.21 ± 20.50	*t* = 0.23	0.82
FVC, %predicted	71.52 ± 19.40	72.14 ± 20.26	69.63 ± 16.81	*t* = 0.53	0.59
DLCO, %predicted	57.62 ± 35.09	58.34 ± 34.40	55.41 ± 37.83	*t* = 0.35	0.73
PO2, (mmHg)	111.04 ± 36.35	111.54 ± 37.63	109.52 ± 32.88	*t* = 0.23	0.82
PCO2, (mmHg)	38.00 ± 4.40	37.96 ± 4.39	38.11 ± 4.52	*t* = −0.14	0.89
PH	7.42 ± 0.05	7.42 ± 0.05	7.43 ± 0.06	*t* = −0.66	0.51
ANA, *n*(%)				*χ*^2^ = 0.61	0.44
−	51 (54.84)	40 (57.14)	11 (47.83)		
+	42 (45.16)	30 (42.86)	12 (52.17)		
Anti-RO52, *n*(%)				*χ*^2^ = 0.67	0.41
−	35 (37.63)	28 (40.00)	7 (30.43)		
+	58 (62.37)	42 (60.00)	16 (69.57)		
Anti-MDA5, *n*(%)				χ^2^ = 0.49	0.49
−	79 (84.95)	61 (87.14)	18 (78.26)		
+	14 (15.05)	9 (12.86)	5 (21.74)		
Anti-Jo1, *n*(%)				χ^2^ = 3.65	0.06
−	67 (72.04)	54 (77.14)	13 (56.52)		
+	26 (27.96)	16 (22.86)	10 (43.48)		
Anti-EJ, *n*(%)				χ^2^ = 0.00	1.00
−	83 (89.25)	62 (88.57)	21 (91.30)		
+	10 (10.75)	8 (11.43)	2 (8.70)		
Anti-PL 7, *n*(%)				χ^2^ = 0.00	0.98
−	83 (89.25)	63 (90.00)	20 (86.96)		
+	10 (10.75)	7 (10.00)	3 (13.04)		
Anti-SRP, *n*(%)				−	0.44
−	91 (97.85)	69 (98.57)	22 (95.65)		
+	2 (2.15)	1 (1.43)	1 (4.35)		
Anti-PMScl75, n(%)				−	1.00
−	91 (97.85)	68 (97.14)	23 (100.00)		
+	2 (2.15)	2 (2.86)	0 (0.00)		
Anti-PMScl100, *n*(%)				−	1.00
−	92 (98.92)	69 (98.57)	23 (100.00)		
+	1 (1.08)	1 (1.43)	0 (0.00)		
Anti-Ku, *n*(%)				−	1.00
−	91 (97.85)	68 (97.14)	23 (100.00)		
+	2 (2.15)	2 (2.86)	0 (0.00)		
Anti-U1RNP, *n*(%)				−	1.00
−	92 (98.92)	69 (98.57)	23 (100.00)		
+	1 (1.08)	1 (1.43)	0 (0.00)		
Anti-SSA, *n*(%)				χ^2^ = 0.04	0.83
−	86 (92.47)	64 (91.43)	22 (95.65)		
+	7 (7.53)	6 (8.57)	1 (4.35)		
Anti-Mi2, *n*(%)				−	0.06
−	91 (97.85)	70 (100.00)	21 (91.30)		
+	2 (2.15)	0 (0.00)	2 (8.70)		
Anti centromere antibody, *n*(%)				−	1.00
−	89 (95.70)	67 (95.71)	22 (95.65)		
+	4 (4.30)	3 (4.29)	1 (4.35)		
Cough, *n*(%)				χ^2^ = 0.53	0.46
NO	23 (24.73)	16 (22.86)	7 (30.43)		
YES	70 (75.27)	54 (77.14)	16 (69.57)		
Expectoration, *n*(%)				χ^2^ = 0.32	0.57
NO	28 (30.11)	20 (28.57)	8 (34.78)		
YES	65 (69.89)	50 (71.43)	15 (65.22)		
Chest tightness, *n*(%)				χ^2^ = 0.09	0.77
NO	30 (32.26)	22 (31.43)	8 (34.78)		
YES	63 (67.74)	48 (68.57)	15 (65.22)		
Fever, *n*(%)				χ^2^ = 0.09	0.76
NO	43 (46.24)	33 (47.14)	10 (43.48)		
YES	50 (53.76)	37 (52.86)	13 (56.52)		
Rash, *n*(%)				χ^2^ = 0.11	0.74
NO	35 (37.63)	27 (38.57)	8 (34.78)		
YES	58 (62.37)	43 (61.43)	15 (65.22)		
Raynaud phenomenon, *n*(%)				χ^2^ = 0.17	0.68
NO	85 (91.40)	63 (90.00)	22 (95.65)		
YES	8 (8.60)	7 (10.00)	1 (4.35)		
Joint pain, *n*(%)				χ^2^ = 0.03	0.85
NO	42 (45.16)	32 (45.71)	10 (43.48)		
YES	51 (54.84)	38 (54.29)	13 (56.52)		
Muscle soreness, *n*(%)				χ^2^ = 2.25	0.13
NO	49 (52.69)	40 (57.14)	9 (39.13)		
YES	44 (47.31)	30 (42.86)	14 (60.87)		
Technician’s Hand, *n*(%)				χ^2^ = 0.47	0.49
NO	72 (77.42)	53 (75.71)	19 (82.61)		
YES	21 (22.58)	17 (24.29)	4 (17.39)		
Reticular shadow, *n*(%)				χ^2^ = 5.16	0.02
NO	30 (32.26)	27 (38.57)	3 (13.04)		
YES	63 (67.74)	43 (61.43)	20 (86.96)		
Nodular shadow, *n*(%)				χ^2^ = 1.44	0.23
NO	59 (63.44)	42 (60.00)	17 (73.91)		
YES	34 (36.56)	28 (40.00)	6 (26.09)		
Honeycomb shadow, *n*(%)				χ^2^ = 0.00	0.98
NO	79 (84.95)	60 (85.71)	19 (82.61)		
YES	14 (15.05)	10 (14.29)	4 (17.39)		
Ground glass opacity, *n*(%)				χ^2^ = 0.19	0.66
NO	76 (81.72)	56 (80.00)	20 (86.96)		
YES	17 (18.28)	14 (20.00)	3 (13.04)		
ILD, *n*(%)				−	0.96
UIP	17 (18.28)	12 (17.14)	5 (21.74)		
NSIP	43 (46.24)	33 (47.14)	10 (43.48)		
OP	29 (31.18)	22 (31.43)	7 (30.43)		
Other	4 (4.30)	3 (4.29)	1 (4.35)		
IIM types				χ^2^ = 1.08	0.58
ASS	55(59.14)	41(58.57)	14(60.87)		
PM	8(8.60)	24(34.29)	6(26.09)		
DM	30(32.26)	5(7.14)	3(13.04)		
Types of hormones				−	0.041
Methylprednisolone	73 (78.49)	59(84.59)	14(60.87)		
Prednisone	18(19.35)	10(14.59)	8(34.78)		
Not used	2 (2.15)	1(1.43)	1(4.35)		
Hormone dosage(mg/d)				χ^2^ = 0.97	0.33
<=40	25 (26.88)	17 (24.29)	8 (34.78)		
>40	68 (73.12)	53 (75.71)	15 (65.22)		

t: *t*-test, χ^2^: Chi-square test, −: Fisher exact, SD: standard deviation.

### Drug use

The mean prednisone equivalent dose in the total cohort was 57.66 ± 33.31 mg/day. The mean dose was 60.68 ± 33.48 mg/day in the non-recurrence group and 48.48 ± 31.75 mg/day in the recurrence group. Although the recurrence group received a lower average dose, the difference was not statistically significant (*p* = 0.13), indicating comparable corticosteroid exposure between the two groups. The non-recurrence group showed a greater tendency to use methylprednisolone, with a slightly higher proportion of patients receiving high doses (>40 mg/day). In contrast, the recurrence group had a relatively higher proportion of patients using prednisone and receiving low doses (≤40 mg/day). At the time of the first recurrence, the average equivalent prednisone dosage required was 32.17 ± 27.68 mg/day. The use of immunosuppressive agents—including hydroxychloroquine, thalidomide, cyclophosphamide, cyclosporine, mycophenolate mofetil, tacrolimus, methotrexate, and tofacitinib—was also assessed. Chi-square and Fisher’s exact tests showed no significant differences in usage proportions between the two groups (all *p* > 0.05), suggesting consistent treatment regimens across the cohort. In terms of the equivalent prednisone dosage during the stable phase, the recurrence group was slightly lower than the non-recurrence group (5.69 ± 2.05 mg/day vs. 5.33 ± 1.74 mg/day), with a statistically significant difference. Regarding the tapering rate, the recurrence group was also slightly lower than the non-recurrence group (4.99 ± 2.52 mg/day/month vs. 4.70 ± 2.08 mg/day/month), but the difference was not statistically significant (*p* = 0.45) (Supplementary Table 2).

### Laboratory indicators of recurrence group

During recurrence episodes, counts of lymphocytes, monocytes, white blood cells, neutrophils, and red blood cells decreased. Among these, only the reduction in white blood cell count reached statistical significance. Platelet count showed an upward trend, although the difference was not statistically significant. C-reactive protein levels decreased during recurrence, with the change approaching statistical significance, suggesting a possible reduction in systemic inflammation. In contrast, ESR and LDH levels increased significantly during recurrence (*p* < 0.05). Serum levels of CK, ALT, and AST also rose during recurrence; however, these changes did not reach statistical significance. Immunoglobulin levels (IgM, IgA, and IgG) and complement C3 levels declined during recurrence, though these decreases were not statistically significant (Supplementary Table 1).

### Single-factor and multi-factor analysis

Univariate Cox regression analysis was conducted to identify factors associated with recurrence. Variables with a *p-*value < 0.1 were subsequently included in the multivariate analysis using a forward selection approach. These included: types of idiopathic inflammatory myopathies (IIMs), positive anti-Ro52 antibody, positive anti-PL7 antibody, elevated white blood cell count, elevated neutrophil count, chest tightness and dyspnea, joint pain, elevated platelet count, elevated aspartate aminotransferase (AST), elevated alanine aminotransferase (ALT), elevated lactate dehydrogenase (LDH), and elevated immunoglobulin A (IgA) (Table 2).

**Table 2:** Univariate Cox regression results of risk factors for recurrence in IIM-ILD patients.

Variables	*β*	S.E	Z	*P*	HR (95%CI)
Sex					
Man					1.00 (Reference)
Women	0.18	0.64	0.28	0.78	1.20 (0.34 ~ 4.21)
IIM types					
ASS					1.00 (Reference)
DM	−1.26	0.59	−2.14	**0.03**	0.28 (0.09 ~ 0.90)
PM	−0.18	0.65	−0.27	0.79	0.84 (0.23 ~ 3.00)
Smoking					
NO					1.00 (Reference)
YES	−0.18	0.64	−0.28	0.78	0.84 (0.24 ~ 2.95)
ANA					
−					1.00 (Reference)
+	−0.17	0.46	−0.38	0.70	0.84 (0.34 ~ 2.06)
Anti-RO52					
−					1.00 (Reference)
+	1.08	0.58	1.87	0.06	2.94 (0.95 ~ 9.13)
Anti-MDA5					
−					1.00 (Reference)
+	−0.93	0.64	−1.45	0.15	0.39 (0.11 ~ 1.38)
Anti-Jo1					
−					1.00 (Reference)
+	0.16	0.44	0.36	0.72	1.17 (0.49 ~ 2.78)
Anti-EJ					
−					1.00 (Reference)
+	0.12	0.75	0.16	0.87	1.13 (0.26 ~ 4.94)
Anti-PL7					
−					1.00 (Reference)
+	2.03	0.78	2.59	**<0.01**	7.62 (1.64 ~ 35.37)
Anti-SSA					
−					1.00 (Reference)
+	−0.69	1.04	−0.67	0.51	0.50 (0.07 ~ 3.83)
Anti-Mi2					
−					1.00 (Reference)
+	−1.30	1.05	−1.24	0.21	0.27 (0.03 ~ 2.12)
Anticentromere antibody					
−					1.00 (Reference)
+	−0.89	1.04	−0.85	0.39	0.41 (0.05 ~ 3.16)
Cough					
NO					1.00 (Reference)
YES	0.14	0.48	0.29	0.78	1.15 (0.44 ~ 2.97)
Expectoration					
NO					1.00 (Reference)
YES	0.03	0.46	0.07	0.94	1.03 (0.42 ~ 2.56)
Chest tightness					
NO					1.00 (Reference)
YES	−1.69	0.54	−3.14	**<0.01**	0.18 (0.06 ~ 0.53)
Fever					
NO					1.00 (Reference)
YES	0.25	0.46	0.54	0.59	1.28 (0.52 ~ 3.16)
Rash					
NO					1.00 (Reference)
YES	−0.57	0.47	−1.22	0.22	0.56 (0.22 ~ 1.42)
Raynaud phenomenon					
NO					1.00 (Reference)
YES	−0.52	1.04	−0.51	0.61	0.59 (0.08 ~ 4.50)
Joint pain					
NO					1.00 (Reference)
YES	−0.98	0.46	−2.12	**0.03**	0.38 (0.15 ~ 0.93)
Musclesoreness					
NO					1.00 (Reference)
YES	−0.06	0.45	−0.14	0.89	0.94 (0.39 ~ 2.25)
Technicians hand					
NO					1.00 (Reference)
YES	0.69	0.57	1.21	0.23	2.00 (0.65 ~ 6.18)
Hydroxychloroquine					
NO					1.00 (Reference)
YES	0.01	0.52	0.02	0.99	1.01 (0.36 ~ 2.79)
Thalidomide					
NO					1.00 (Reference)
YES	2.37	1.22	1.93	0.05	10.66 (0.97 ~ 117.61)
Cyclophosphamide					
NO					1.00 (Reference)
YES	−0.80	0.56	−1.41	0.16	0.45 (0.15 ~ 1.36)
Cyclosporine					
NO					1.00 (Reference)
YES	0.57	0.64	0.89	0.37	1.77 (0.51 ~ 6.19)
Tacrolimus					
NO					1.00 (Reference)
YES	0.63	0.65	0.98	0.33	1.89 (0.53 ~ 6.71)
Tofacitinib					
NO					1.00 (Reference)
YES	0.80	1.06	0.76	0.45	2.23 (0.28 ~ 17.91)
Mycophenolate mofetil					
NO					1.00 (Reference)
YES	0.97	0.77	1.26	0.21	2.64 (0.58 ~ 11.96)
Reticular shadow					
NO					1.00 (Reference)
YES	−0.07	0.63	−0.11	0.91	0.93 (0.27 ~ 3.21)
Nodular shadow					
NO					1.00 (Reference)
YES	−0.06	0.49	−0.12	0.90	0.94 (0.36 ~ 2.44)
Honeycomb shadow					
NO					1.00 (Reference)
YES	0.26	0.57	0.46	0.64	1.30 (0.43 ~ 3.97)
Ground glass opacity					
NO					1.00 (Reference)
YES	0.24	0.64	0.37	0.71	1.27 (0.36 ~ 4.41)
ILD					
UIP					1.00 (Reference)
NSIP	−0.16	0.56	−0.28	0.78	0.85 (0.28 ~ 2.58)
OP	−0.02	0.60	−0.03	0.98	0.98 (0.30 ~ 3.19)
Other	−0.32	1.11	−0.29	0.77	0.73 (0.08 ~ 6.46)
Withdrawal					
NO					1.00 (Reference)
Yes	−0.06	0.45	−0.14	0.89	0.94 (0.39 ~ 2.28)
Time	−0.01	0.01	−1.37	0.17	0.99 (0.97 ~ 1.01)
Age	0.03	0.02	1.29	0.20	1.03 (0.98 ~ 1.08)
Prednisone	0.01	0.01	0.95	0.34	1.01 (0.99 ~ 1.02)
Leukocyte	0.10	0.06	1.69	0.09	1.10 (0.98 ~ 1.24)
Monocyte	−0.03	0.81	−0.04	0.97	0.97 (0.20 ~ 4.77)
Neutrophils	0.11	0.06	1.71	0.09	1.12 (0.98 ~ 1.27)
Lymphocyte	0.16	0.30	0.52	0.60	1.17 (0.65 ~ 2.10)
NLR	0.05	0.04	1.21	0.22	1.05 (0.97 ~ 1.13)
MLR	−0.48	1.20	−0.40	0.69	0.62 (0.06 ~ 6.43)
SIRI	0.09	0.08	1.08	0.28	1.09 (0.93 ~ 1.27)
S11	0.00	0.00	1.61	0.11	1.00 (1.00 ~ 1.00)
Erythrocyte	0.13	0.43	0.30	0.76	1.14 (0.49 ~ 2.68)
Platelet	0.01	0.00	2.17	**0.03**	1.01 (1.01 ~ 1.01)
ESR	0.01	0.01	0.40	0.69	1.01 (0.98 ~ 1.03)
CRP	0.04	0.02	1.54	0.12	1.04 (0.99 ~ 1.08)
CK	0.00	0.00	1.36	0.17	1.00 (1.00 ~ 1.00)
AST	0.01	0.00	1.81	0.07	1.01 (1.00 ~ 1.01)
ALT	0.01	0.00	1.75	0.08	1.01 (1.00 ~ 1.02)
LDH	0.00	0.00	1.70	0.09	1.00 (1.00 ~ 1.00)
IgG	0.08	0.07	1.19	0.23	1.08 (0.95 ~ 1.23)
IgM	−0.00	0.27	−0.01	0.99	1.00 (0.58 ~ 1.70)
IgA	0.49	0.21	2.35	**0.02**	1.63 (1.08 ~ 2.44)
C3	−0.44	0.91	−0.49	0.63	0.64 (0.11 ~ 3.82)
FEV1	0.01	0.01	0.72	0.47	1.01 (0.98 ~ 1.04)
FVC	0.02	0.02	1.22	0.22	1.02 (0.99 ~ 1.05)
DLCO	0.00	0.01	0.71	0.48	1.00 (0.99 ~ 1.02)
PO2	−0.01	0.01	−1.29	0.20	0.99 (0.97 ~ 1.01)
PCO2	0.08	0.06	1.39	0.17	1.08 (0.97 ~ 1.21)
PH	3.33	4.03	0.82	0.41	27.82 (0.01 ~ 75481.07)

HR: Hazards Ratio, CI: Confidence Interval. Bold values indicate: *p* < 0.05.

**Figure 2:**
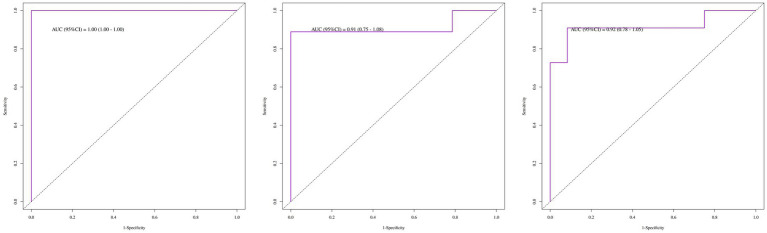
ROC curves for the recurrence risk of patients with IIM-ILD (1-year, 2-year, 3-year).

**Figure 3:**
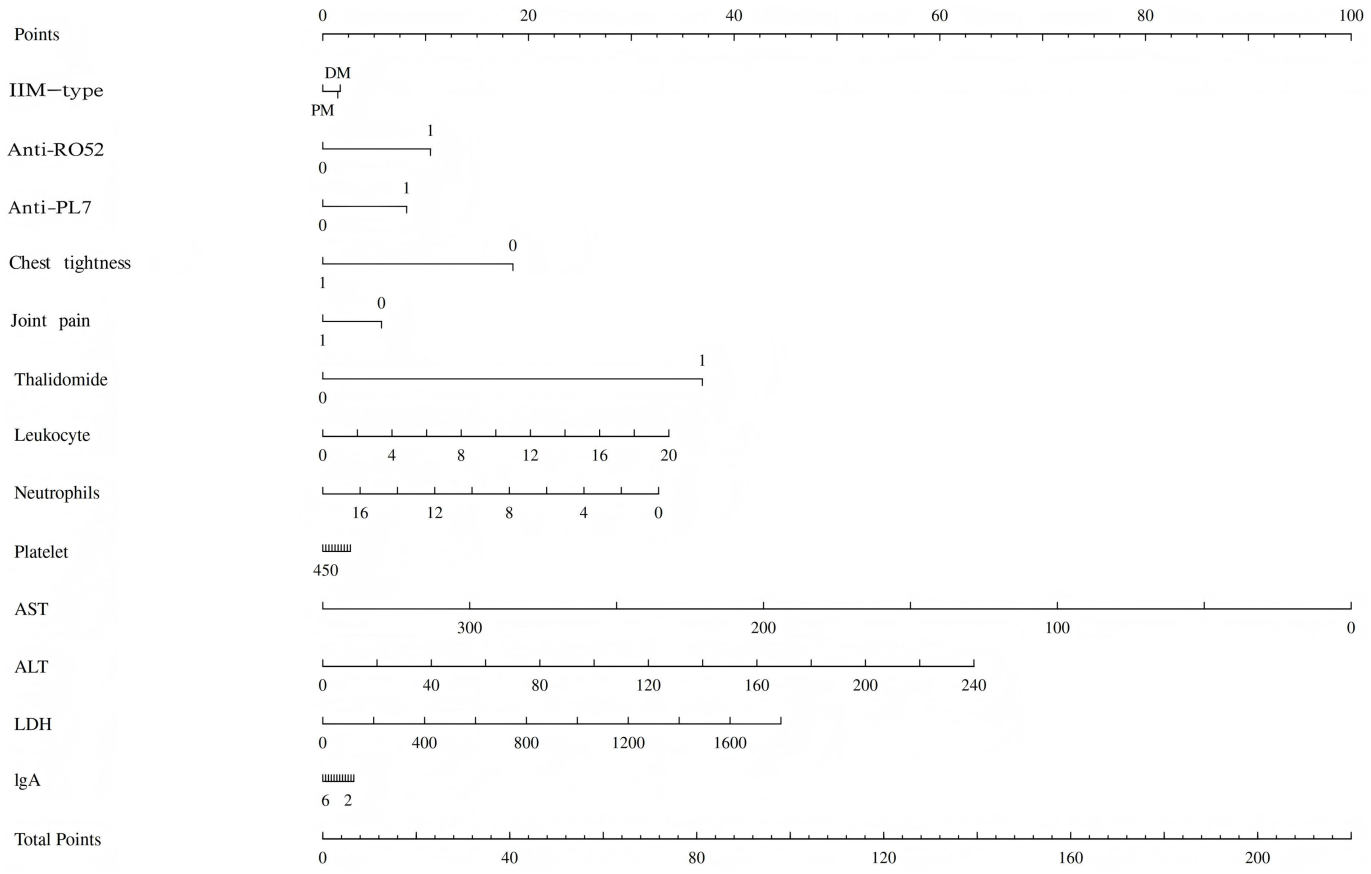
Nomogram of risk factors for recurrence risk of patients with IIM-ILD.

The multivariate Cox regression analysis revealed that positive anti-Ro52 antibody, positive anti-PL7 antibody, elevated ALT, elevated white blood cell count, and elevated LDH were high-risk factors for recurrence, while joint pain, elevated AST, elevated neutrophil count, and chest tightness were protective factors. Except for chest tightness, none of the other factors retained statistical significance in the final model (Table 3). The proportional hazards assumption was tested for all covariates and was satisfied (*p* > 0.05 for both individual and global tests). The model demonstrated strong performance: likelihood ratio test = 42.22, Wald test = 13.97, score (log-rank) test = 35.87, and C-index = 0.91 (standard error = 0.04), indicating excellent discriminative ability. The area under the ROC curve (AUC) for recurrence prediction was:1-year: 1.00 (95% CI: 1.00–1.00), 2-year: 0.91 (95% CI: 0.75–1.08), 3-year: 0.92 (95% CI: 0.78–1.05) (Figure 2). Calibration curves and the C-index confirmed the high predictive accuracy of the model (Supplementary Figure 2). Based on the final multivariate Cox regression, nomograms were constructed to estimate recurrence risk at 1, 2, and 3 years (Figure 3). Each nomogram assigns a weighted score to each predictor, with longer line segments indicating a greater influence on outcome risk. Decision curve analysis (DCA) was also conducted for each time point, demonstrating favorable clinical utility.

**Table 3:** Multivariate Cox regression results of risk factors for recurrence in IIM-ILD patients.

Variables	*β*	S.E	*Z*	*p*	HR (95%CI)
IIM types					
ASS					1.00 (Reference)
DM	−0.96	2.48	−0.39	0.70	0.38 (0.00 ~ 49.12)
PM	−0.71	1.18	−0.60	0.55	0.49 (0.05 ~ 4.94)
Anti-RO52					
–					1.00 (Reference)
+	3.68	1.94	1.89	0.06	39.66 (0.88 ~ 1792.84)
Anti-PL7					
–					1.00 (Reference)
+	3.62	2.03	1.78	0.07	37.22 (0.70 ~ 1989.17)
Chest tightness					
NO					1.00 (Reference)
YES	−5.76	2.38	−2.42	**0.02**	0.00 (0.00 ~ 0.34)
Joint pain					
NO					1.00 (Reference)
YES	−1.70	1.38	−1.23	0.22	0.18 (0.01 ~ 2.76)
Thalidomide					
−					1.00 (Reference)
+	11.19	4.30	2.60	**<0.01**	72180.74 (15.80 ~ 329716.51)
Leukocyte	0.40	0.56	0.71	0.48	1.49 (0.50 ~ 4.44)
Neutrophils	−0.44	0.58	−0.76	0.45	0.64 (0.20 ~ 2.02)
Platelet	−0.00	0.01	−0.15	0.88	1.00 (0.99 ~ 1.01)
AST	−0.08	0.05	−1.57	0.12	0.93 (0.84 ~ 1.02)
ALT	0.07	0.04	1.64	0.10	1.08 (0.99 ~ 1.17)
LDH	0.01	0.01	1.05	0.29	1.01 (0.99 ~ 1.02)
IgA	−0.27	0.75	−0.35	0.72	0.77 (0.18 ~ 3.33)

HR: Hazards Ratio, CI: Confidence Interval. Bold values indicate: *p* < 0.05.

### Sensitivity analysis

We excluded patients with a follow-up duration significantly shorter than the recurrence time, those lacking pulmonary function data, and those who discontinued treatment during the course of therapy. Three predictive models were re-established to test the stability of the models (Supplementary Table 3). The results confirmed that there were no significant changes in key variables, ROC, or C-index, indicating that the models are relatively stable.

### Recurrence Kaplan–Meier curves

**Figure 4:**
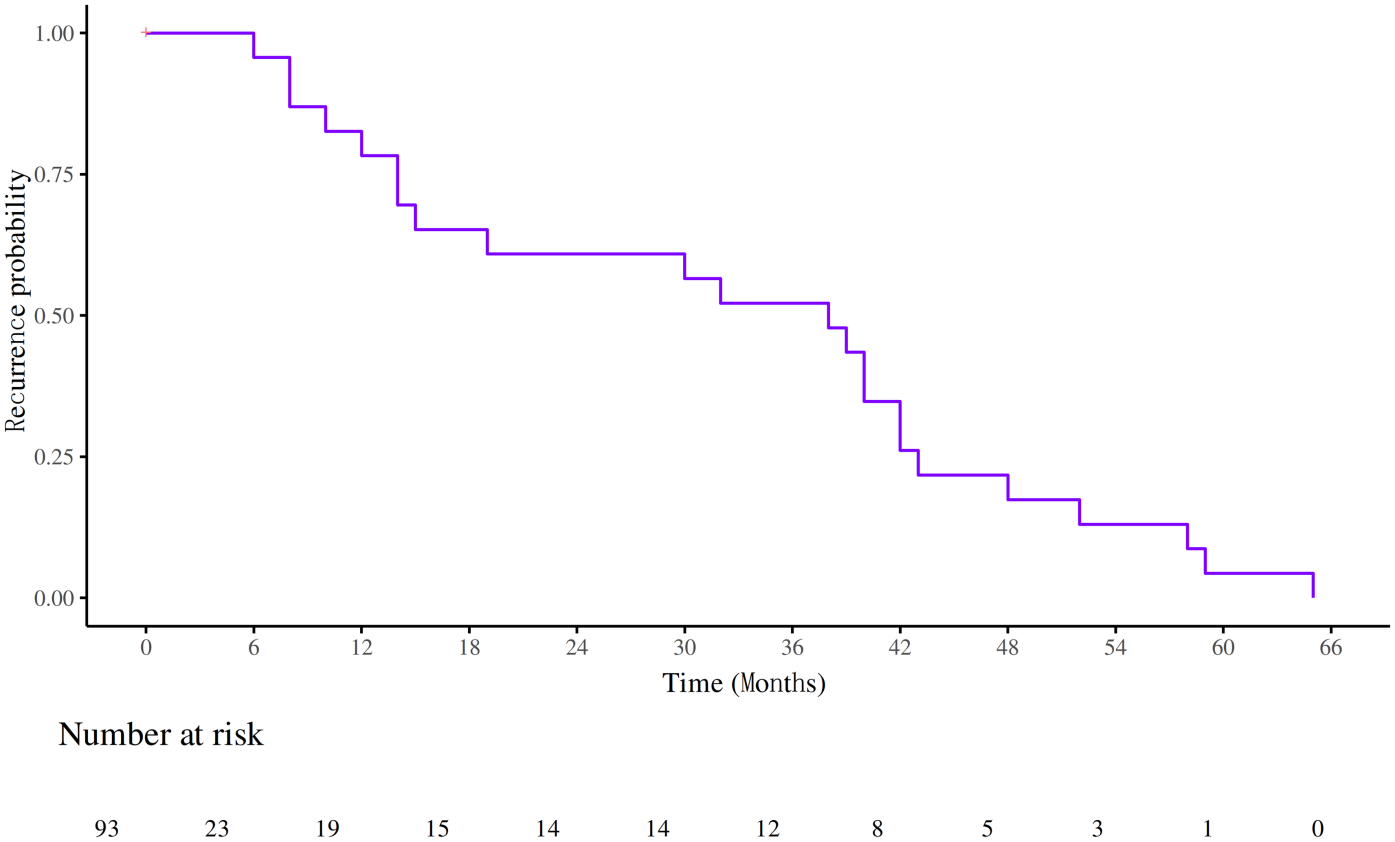
Survival curves of recurrence in patients with IIM-ILD.

Among the 93 patients with IIM-ILD, 23 experienced recurrences, yielding a recurrence rate of 23.7%. The median time to recurrence was 38 months. The identified causes of recurrence included infection (3 cases), drug withdrawal (6 cases), drug modification (1 case), and unknown causes (13 cases) (Figure 4). Kaplan–Meier survival curves were generated for patients stratified by Anti-RO52 and Anti-PL7 antibody status. Both subgroup analyses showed significant differences in recurrence-free survival (log-rank *p* < 0.05). Patients were further classified into high- and low-risk categories according to the median nomogram score. Kaplan–Meier analysis demonstrated that those in the high-risk group had a notably shorter recurrence-free survival than those in the low-risk group (Supplementary Figure 3).

## Discussion

Our study is currently the largest sample-size research on the recurrence factors of IIM - ILD. Our study has confirmed that positive Anti-RO52, positive Anti-PL7, elevated white blood cells, elevated ALT, elevated LDH, and high IgA may be important factors leading to the exacerbation and recurrence of ILD. Meanwhile, we found that 23 out of 93 patients experienced recurrence, and approximately 26.1% of the recurrence cases were due to drug withdrawal or drug change. However, due to missing data, we were unable to further analyze which drug has a relatively significant impact on recurrence.

Anti-Ro52 and Anti-PL7 are two common autoantibodies found in patients with interstitial lung disease (ILD). Several previous studies have demonstrated associations between these antibodies and poor ILD prognosis, including outcomes such as death, acute exacerbation, and recurrence (27). Anti-Ro52 is the most frequently detected antinuclear antibody in idiopathic inflammatory myopathies (IIM) (29). A meta-analysis published in 2023 showed that positive anti-Ro52 is associated with a higher frequency of ILD diagnosis in various autoimmune diseases and is also related to more severe lung lesions (RP–ILD) (30). Fei Xiao et al. developed a prediction model to assess the risk of severe ILD within 6 months among MDA5-positive DM-ILD patients, using quantitative analysis of chest HRCT images. Their results identified anti-Ro52 positivity as the only independent risk factor for severe ILD. The resulting STRAD-Ro52 model demonstrated strong predictive performance for poor IIM-ILD outcomes (23). However, some studies have reported no significant difference in the positivity rate or titer of anti-Ro52 between recurrence and non-recurrence groups (31, 32). Our findings support a significant positive correlation between anti-Ro52 and disease recurrence, which may help reconcile these conflicting reports. Regarding Anti-PL7, research has indicated that its positive rate is higher in ILD patients than in those without ILD (33). A retrospective study by Japanese researchers found a recurrence rate of 16.7% for the anti-synthetase syndrome (ASS) in patients positive for anti-PL7. Xi Zhan et al. conducted a retrospective study on the clinical data of 108 ARS-positive ILD patients and found that most ASSD - ILD patients had a positive response to steroid treatment, with or without the use of immunosuppressants. The incidence of the UIP pattern was the highest in the PL–7 (+) group, and the response to treatment was significantly lower (34). Some studies have also confirmed that positive anti-PL7 is associated with later disease deterioration (35). However, there is still a lack of research on the relationship between anti-PL7 and recurrence. Our results can only cautiously support previous research findings, and the specific relationship between this antibody and recurrence requires further study. As a known antibody closely associated with poor prognosis in IIM-ILD, the anti-MDA5 antibody showed no correlation in our study results. We believe this may be related to the following reasons: 1. The number of patients positive for anti-MDA5 antibody is small, and the incidence of recurrence among them is even lower, resulting in an unobvious correlation. 2. The number of patients included in this study is too small, which may lead to significant bias. 3. The patients involved in this study include different disease subtypes such as DM, PM, and ASS. Various coexisting antibodies or early treatment interventions may indirectly mask the independent association between anti-MDA5 antibody and adverse events.

Elevated neutrophil levels also appear to play a significant role in the poor prognosis of IIM-ILD. Several studies have shown that neutrophil-related inflammatory markers are closely associated with adverse IIM-ILD outcomes (36, 37). During the inflammatory response in dermatomyositis, various cytokines, and growth factors prolong neutrophil survival and enhance their activation. In turn, neutrophil-secreted cytokines further amplify inflammation, while the systemic inflammatory state contributes to lymphocyte apoptosis. However, our study suggests that a higher neutrophil count has an opposite effect on the recurrence of IIM-ILD in patients. This phenomenon is quite interesting. Given the paucity of research on the recurrence of IIM-ILD, our explanation for this phenomenon lacks theoretical support, making it essential to conduct more relevant studies. Together, these mechanisms result in significantly elevated neutrophil counts in PM/DM patients (36). Nevertheless, further high-quality studies are needed to confirm their predictive value for disease recurrence.

Our study also found that elevated white blood cell count may be closely associated with the recurrence of IIM-ILD. Previous studies have confirmed that baseline white blood cell count is closely related to an increased risk of death in IIM-ILD patients (38, 39). This phenomenon may be associated with decreased lung function, pulmonary inflammatory environment, and other factors. Our study suggests that white blood cell count could be a new prognostic predictor for patients with IIM-ILD.

AST and ALT, commonly used liver function markers, are often employed to evaluate the severity and prognosis of liver diseases such as cancer and hepatitis. In our study, elevated AST and ALT were significantly associated with recurrence in IIM-ILD (22). Some studies have confirmed that the DRR index related to AST and ALT is associated with death, exacerbation, and readmission in IIM–ILD patients (40). The observed elevations in AST and ALT may reflect increased right heart afterload due to hypoxia and muscle damage from PM/DM, suggesting a pathophysiological link between these enzymes and IIM-ILD (40, 41). Paying attention to these indicators may be of great significance for predicting the poor prognosis of ILD. The relationship between high IgA and the prognosis of IIM–ILD remains unclear, but our study suggests that hypergammaglobulinemia may be related to the recurrence and poor prognosis of IIM-ILD. The immunological mechanisms involved require more basic and clinical research to be revealed. Given the high risk of poor prognosis in IIM-ILD, early risk assessment is crucial upon diagnosis. The prediction model proposed in our study can aid in evaluating disease severity, guiding clinical decisions, and enabling personalized treatment strategies.

However, our study has several limitations. First, it is a single-center retrospective study, which may introduce selection bias. The included patients may not represent the broader target population. Additionally, as a retrospective analysis, it is difficult to establish causal relationships, and various confounding factors may have influenced the outcomes. These limitations reduce the generalizability of our findings to other populations, regions, or healthcare settings. Regarding the definition of recurrence, it varies across different studies; perhaps the initiation of anti-fibrotic therapy and an increase in corticosteroid dosage could both be considered as indicators of recurrence (42). Moreover, key variables such as ferritin and pulmonary function were excluded due to substantial data loss, possibly affecting the completeness of our conclusions. Finally, due to the rarity of IIM-ILD and the small sample size, the reliability of our findings is somewhat limited. Our study lacks information on myopathy severity as assessed by the MMT-8 score, pulmonary hypertension, and the severity of skin involvement (CDASI score). This may have an impact on the clarification of disease characteristics and the prediction of recurrence, among other aspects. In the future, it will be necessary to more meticulously include the characteristics of the study population to enhance the comprehensiveness of the research. We hope that more high-quality prospective studies will be conducted in the future, so as to further improve the clinical diagnosis and treatment level of IIM-ILD.

## Conclusion

In conclusion, based on baseline clinical and laboratory indicators, we have developed a tool with good predictive ability for the recurrence of IIM-ILD patients. It has identified several factors closely related to the risk of recurrence, such as anti-RO52 antibody and anti-PL7 antibody. This is of high clinical significance for the treatment of the disease.

## Data Availability

The raw data supporting the conclusions of this article will be made available by the authors, without undue reservation.

## Glossary

IIMs: Idiopathic inflammatory myopathies
DM: Dermatomyositis
ILD: Interstitial lung disease
IIM-ILD: Idiopathic Inflammatory Myopathies-Associated Interstitial Lung Disease
PM: Polymyositis
PM/DM: Polymyositis/dermatomyositis
NSIP: Nonspecific interstitial pneumonia
OP: Organizing pneumonia
UIP: Usual interstitial pneumonia
HRCT: High-resolution computed tomography
FVC: Forced vital capacity
DLCO: Diffusing capacity for carbon monoxide
FEV1: Forced expiratory volume in one second
NLR: Neutrophil-to-lymphocyte ratio
MLR: Monocyte-to-lymphocyte ratio
SII: Systemic immune-inflammation index
SIRI: Systemic inflammation response index
ROC: Receiver operating characteristic
AUC: Area under the curve
CI: Confidence interval
DCA: Decision curve analysis
LDH: Lactate dehydrogenase
CRP: C-reactive protein
IgG: Immunoglobulin G
IgA: Immunoglobulin A
IgM: Immunoglobulin M
ESR: Erythrocyte sedimentation rate
CK: Creatine kinase
PaO₂: Partial pressure of oxygen in arterial blood
PaCO₂: Partial pressure of carbon dioxide in arterial blood
ALT: Alanine aminotransferase
AST: Aspartate aminotransferase
EULAR/ACR: European League Against Rheumatism/American College of Rheumatology
PPF: Progressive pulmonary fibrosis
ASS: Anti-synthetase syndrome
ASSD-ILD: Anti-synthetase syndrome-associated interstitial lung disease
ARS: Anti-aminoacyl-tRNA synthetase
MDA5: Melanoma differentiation-associated gene 5
HR: Hazard Ratio
SD: Standard deviation
IQR: Interquartile ranges

## References

[1] Lundberg IE Fujimoto M Vencovsky J Aggarwal R Holmqvist M Christopher-Stine L. Idiopathic inflammatory myopathies. Nat Rev Dis Primers. (2021) 7:86. doi: 10.1038/s41572-021-00321-x 34857798

[2] Bohan A Peter JB. Polymyositis and dermatomyositis (second of two parts). N Engl J Med. (1975) 292:403–7. doi: 10.1056/nejm197502202920807, PMID: 1089199

[3] Lega JC Reynaud Q Belot A Fabien N Durieu I Cottin V. Idiopathic inflammatory myopathies and the lung. Eur Respir Rev. (2015) 24:216–38. doi: 10.1183/16000617.00002015, PMID: 26028634 PMC9487811

[4] Hervier B Devilliers H Stanciu R Meyer A Uzunhan Y Masseau A. Hierarchical cluster and survival analyses of antisynthetase syndrome: phenotype and outcome are correlated with anti-tRNA synthetase antibody specificity. Autoimmun Rev. (2012) 12:210–7. doi: 10.1016/j.autrev.2012.06.006, PMID: 22771754

[5] Li S Sun Y Shao C Huang H Wang Q Xu K. Prognosis of adult idiopathic inflammatory myopathy-associated interstitial lung disease: a retrospective study of 679 adult cases. Rheumatology (Oxford). (2021) 60:1195–204. doi: 10.1093/rheumatology/keaa372, PMID: 32894294

[6] Lei L Ma Z Ma X Pan D Chen Z Qin F. An observational study on the clinical characteristics and prognosis of patients with interstitial lung disease secondary to dermatomyositis and Antisynthetase syndrome. Int J Rheumatol. (2024) 2024:9679944. doi: 10.1155/2024/9679944, PMID: 39364301 PMC11449546

[7] Takei R Yamano Y Kataoka K Yokoyama T Matsuda T Kimura T. Predictive factors for the recurrence of anti-aminoacyl-tRNA synthetase antibody-associated interstitial lung disease. Respir Investig. (2020) 58:83–90. doi: 10.1016/j.resinv.2019.10.004, PMID: 31813784

[8] Morina G Sambataro D Libra A Palmucci S Colaci M La Rocca G. Recognition of idiopathic inflammatory myopathies underlying interstitial lung diseases. Diagnostics. (2025) 15:275. doi: 10.3390/diagnostics15030275, PMID: 39941205 PMC11817385

[9] Okabayashi H Nakashima S Fujino K Imai M Hamada S Masunaga A. Tension pneumomediastinum in anti-MDA5 antibody-positive dermatomyositis-associated interstitial lung disease: a case report and literature review. Intern Med. (2024) 63:3221–6. doi: 10.2169/internalmedicine.3418-23, PMID: 38569903 PMC11671188

[10] Ma X Chen Z Hu W Guo Z Wang Y Kuwana M. Clinical and serological features of patients with dermatomyositis complicated by spontaneous pneumomediastinum. Clin Rheumatol. (2016) 35:489–93. doi: 10.1007/s10067-015-3001-3, PMID: 26149923

[11] Cottin V Nunes H Mouthon L Gamondes D Lazor R Hachulla E. Combined pulmonary fibrosis and emphysema syndrome in connective tissue disease. Arthritis Rheum. (2011) 63:295–304. doi: 10.1002/art.30077, PMID: 20936629

[12] Hallowell RW Danoff SK. Diagnosis and management of myositis-associated lung disease. Chest. (2023) 163:1476–91. doi: 10.1016/j.chest.2023.01.031, PMID: 36764512

[13] Fujisawa T Hozumi H Kamiya Y Kaida Y Akamatsu T Kusagaya H. Prednisolone and tacrolimus versus prednisolone and cyclosporin a to treat polymyositis/dermatomyositis-associated ILD: a randomized, open-label trial. Respirology (Carlton, Vic). (2021) 26:370–7. doi: 10.1111/resp.13978, PMID: 33179395

[14] Go DJ Park JK Kang EH Kwon HM Lee YJ Song YW. Survival benefit associated with early cyclosporine treatment for dermatomyositis-associated interstitial lung disease. Rheumatol Int. (2016) 36:125–31. doi: 10.1007/s00296-015-3328-8, PMID: 26223808

[15] Lian L Li M Li Y Wang K Xu S. Combination therapy of tacrolimus, high doses of glucocorticosteroids, and cyclophosphamide against existing historical treatment for patients in severe conditions of interstitial lung diseases complicated with dermatomyositis: a retrospective analysis. Medicine. (2022) 101:e29108. doi: 10.1097/md.0000000000029108, PMID: 35713427 PMC9276089

[16] Sharma N Putman MS Vij R Strek ME Dua A. Myositis-associated interstitial lung disease: predictors of failure of conventional treatment and response to tacrolimus in a US cohort. J Rheumatol. (2017) 44:1612–8. doi: 10.3899/jrheum.161217, PMID: 28864644

[17] Yoshifuji H Fujii T Kobayashi S Imura Y Fujita Y Kawabata D. Anti-aminoacyl-tRNA synthetase antibodies in clinical course prediction of interstitial lung disease complicated with idiopathic inflammatory myopathies. Autoimmunity. (2006) 39:233–41. doi: 10.1080/08916930600622884 16769657

[18] Kurita T Yasuda S Oba K Odani T Kono M Otomo K. The efficacy of tacrolimus in patients with interstitial lung diseases complicated with polymyositis or dermatomyositis. Rheumatology (Oxford). (2015) 54:1536. doi: 10.1093/rheumatology/kev192, PMID: 26081349

[19] Nakazawa M Kaneko Y Takeuchi T. Risk factors for the recurrence of interstitial lung disease in patients with polymyositis and dermatomyositis: a retrospective cohort study. Clin Rheumatol. (2018) 37:765–71. doi: 10.1007/s10067-017-3854-8, PMID: 28975463

[20] Janardana R Sangeetha KN Bhat V Balakrishnan D Raj JM Pinto B. Long term outcomes in idiopathic inflammatory myositis: an observational epidemiologic study over 15 years. Mediterranean J Rheumatol. (2023) 34:513–24. doi: 10.31138/mjr.280823.lto, PMID: 38282927 PMC10815524

[21] Gui X Li W Yu Y Zhao T Jin Z Meng K. Prediction model for the pretreatment evaluation of mortality risk in anti-melanoma differentiation-associated gene 5 antibody-positive dermatomyositis with interstitial lung disease. Front Immunol. (2022) 13:978708. doi: 10.3389/fimmu.2022.978708, PMID: 36211445 PMC9539924

[22] Niu Q Zhao LQ Ma WL Xiong L Wang X-r He X-l. A new predictive model for the prognosis of MDA5(+) DM-ILD. Front Med. (2022) 9:908365. doi: 10.3389/fmed.2022.908365, PMID: 35783655 PMC9240232

[23] Xiao F Chen F Li D Zheng S Liang X Wu J. Severe interstitial lung disease risk prediction in anti-melanoma differentiation-associated protein 5 positive dermatomyositis: the STRAD-Ro52 model. Ann Med. (2025) 57:2440621. doi: 10.1080/07853890.2024.2440621, PMID: 39697063 PMC11660418

[24] Lundberg IE Tjärnlund A Bottai M Werth VP Pilkington C Visser M. 2017 European league against rheumatism/American College of Rheumatology classification criteria for adult and juvenile idiopathic inflammatory myopathies and their major subgroups. Ann Rheum Dis. (2017) 76:1955–64. doi: 10.1136/annrheumdis-2017-211468, PMID: 29079590 PMC5736307

[25] Connors GR Christopher-Stine L Oddis CV Danoff SK. Interstitial lung disease associated with the idiopathic inflammatory myopathies: what progress has been made in the past 35 years? Chest. (2010) 138:1464–74. doi: 10.1378/chest.10-0180 21138882

[26] Schaefer-Prokop C Prokop M Fleischmann D Herold C. High-resolution CT of diffuse interstitial lung disease: key findings in common disorders. Eur Radiol. (2001) 11:373–92. doi: 10.1007/s003300000648, PMID: 11288840

[27] Raghu G Remy-Jardin M Richeldi L Thomson CC Inoue Y Johkoh T. Idiopathic pulmonary fibrosis (an update) and progressive pulmonary fibrosis in adults: an official ATS/ERS/JRS/ALAT clinical practice guideline. Am J Respir Crit Care Med. (2022) 205:e18–47. doi: 10.1164/rccm.202202-0399ST, PMID: 35486072 PMC9851481

[28] Chen H Liu H Lyu W Liu Y Huang M Zhang Y. An observational study of clinical recurrence in patients with interstitial lung disease related to the antisynthetase syndrome. Clin Rheumatol. (2023) 42:711–20. doi: 10.1007/s10067-022-06424-4, PMID: 36334174

[29] Xu A Ye Y Fu Q Lian X Chen S Guo Q. Prognostic values of anti-Ro52 antibodies in anti-MDA5-positive clinically amyopathic dermatomyositis associated with interstitial lung disease. Rheumatology (Oxford). (2021) 60:3343–51. doi: 10.1093/rheumatology/keaa786, PMID: 33331866

[30] Nayebirad S Mohamadi A Yousefi-Koma H Javadi M Farahmand K Atef-Yekta R. Association of anti-Ro52 autoantibody with interstitial lung disease in autoimmune diseases: a systematic review and meta-analysis. BMJ Open Respir Res. (2023) 10:e002076. doi: 10.1136/bmjresp-2023-002076, PMID: 38030264 PMC10689422

[31] Liu Y Liu X Xie M Chen Z He J Wang Z. Clinical characteristics of patients with anti-EJ antisynthetase syndrome associated interstitial lung disease and literature review. Respir Med. (2020) 165:105920. doi: 10.1016/j.rmed.2020.105920, PMID: 32174452

[32] Matsuda S Kotani T Oe K Okazaki A Kiboshi T Suzuka T. Poor prognostic factors for relapse of interstitial lung disease with anti-aminoacyl-tRNA synthetase antibodies after combination therapy. Front Immunol. (2024) 15:1407633. doi: 10.3389/fimmu.2024.1407633, PMID: 39346900 PMC11427292

[33] Huang HL Lin WC Lin PY Weng MY Sun YT. The significance of myositis autoantibodies in idiopathic inflammatory myopathy concomitant with interstitial lung disease. Neurol Sci. (2021) 42:2855–64. doi: 10.1007/s10072-020-04911-7, PMID: 33211209

[34] Zhan X Yan W Wang Y Li Q Shi X Gao Y. Clinical features of anti-synthetase syndrome associated interstitial lung disease: a retrospective cohort in China. BMC Pulm Med. (2021) 21:57. doi: 10.1186/s12890-021-01399-5, PMID: 33579248 PMC7881640

[35] Fujisawa T Hozumi H Kono M Enomoto N Nakamura Y Inui N. Predictive factors for long-term outcome in polymyositis/dermatomyositis-associated interstitial lung diseases. Respir Investig. (2017) 55:130–7. doi: 10.1016/j.resinv.2016.09.006, PMID: 28274528

[36] Li M Yan S Dong R Xiang W Ma Z Yang Q. Elevated platelet-to-lymphocyte ratio and neutrophil-to-lymphocyte ratio in patients with polymyositis/dermatomyositis: a retrospective study. Clin Rheumatol. (2023) 42:1615–24. doi: 10.1007/s10067-023-06542-7, PMID: 36781682

[37] Tian Y He P Ren L Xin H Xi B Zou R. Dynamic change of lymphocytes associated with short-term prognosis in anti-MDA5-positive dermatomyositis with interstitial lung disease: a multicenter retrospective study. Clin Rheumatol. (2024) 43:3399–408. doi: 10.1007/s10067-024-07110-3, PMID: 39292419 PMC11489275

[38] Li Y Liu X Tian M Zou R Gao Y Huang M. Soluble CD206 levels correlate with disease deterioration and predict prognosis of anti-MDA5 antibody-positive dermatomyositis related interstitial lung disease. Clin Respir J. (2023) 17:507–15. doi: 10.1111/crj.13616, PMID: 37041007 PMC10265147

[39] Yang Y Li Y Yuan W Zhang S He X Ji J. Risk factors for mortality in anti-MDA5 antibody-positive dermatomyositis with interstitial lung disease: a systematic review and meta-analysis. Front Immunol. (2025) 16:1628748. doi: 10.3389/fimmu.2025.1628748, PMID: 40746563 PMC12310698

[40] Li R Zhu WJ Wang F Tang X Luo F. AST/ALT ratio as a predictor of mortality and exacerbations of PM/DM-ILD in 1 year-a retrospective cohort study with 522 cases. Arthritis Res Ther. (2020) 22:202. doi: 10.1186/s13075-020-02286-w, PMID: 32950060 PMC7502203

[41] Dufour DR Lott JA Nolte FS Gretch DR Koff RS Seeff LB. Diagnosis and monitoring of hepatic injury. I. Performance characteristics of laboratory tests. Clin Chem. (2000) 46:2027–49. doi: 10.1093/clinchem/46.12.2027, PMID: 11106349

[42] Wells AU Flaherty KR Brown KK Inoue Y Devaraj A Richeldi L. Nintedanib in patients with progressive fibrosing interstitial lung diseases-subgroup analyses by interstitial lung disease diagnosis in the INBUILD trial: a randomised, double-blind, placebo-controlled, parallel-group trial. Lancet Respir Med. (2020) 8:453–60. doi: 10.1016/s2213-2600(20)30036-9, PMID: 32145830

