# Supplementary material for: Clinical features and recurrence risk prediction model in patients with idiopathic inflammatory myopathies-associated interstitial lung disease: a retrospective study from Ningbo
Source: Front Med (Lausanne). 2025 Sep 10;12:1666332. doi: 10.3389/fmed.2025.1666332 (PMC12457148; doi:10.3389/fmed.2025.1666332)

**eTable 1.Comparison of Laboratory Indicators between the Baseline and the Time of Recurrence in the Recurrent Population**

| **Variables** | **Baseline** | **Ruccrence** | **P** |
| --- | --- | --- | --- |
| **Iymphocyte,(× 10^9/L )** | 1.38 ± 0.92 | 1.22 ± 0.83 | 0.539 |
| **Monocyte, (× 10^9/L)** | 0.80 ± 0.67 | 0.54 ± 0.26 | 0.101 |
| **CRP, (× 10^9/L )** | 9.43 ± 12.1 | 6.40 ± 9.97 | 0.106 |
| **Creatine kinase,(U/L)** | 177.96 ± 342.39 | 518.17 ± 1514.59 | 0.300 |
| **ALT, (U/L)** | 32.04 ± 28.27 | 52.09 ± 56.501 | 0.076 |
| **AST, (U/L)** | 32.35 ± 24.53 | 64.43 ± 80.86 | 0.152 |
| **IgM, (g/L)** | 1.63 ± 1.00 | 1.62 ± 0.94 | 0.953 |
| **IgA, (g/L)** | 2.44 ± 1.63 | 2.33 ± 1.14 | 0.805 |
| **Leukocyte, (× 10^9/L)** | 8.83 (6.67, 10.34) | 6.67 (4.89, 9.56) | 0.042 |
| **Neutrophils, (× 10^9/L)** | 6.20 (4.15, 8.35) | 4.90 (4.07, 6.80) | 0.201 |
| **Erythrocyte, (× 10^9/L)** | 4.33 (4.04, 4.54) | 4.19 (3.88, 4.69) | 0.503 |
| **Platelet, (× 10^9/L)** | 249.00 (215.50, 281.50) | 265.00 (196.00, 311.50) | 0.574 |
| **ESR, (mm/h)** | 23.00 (12.00, 35.50) | 25.00 (18.50, 37.00) | <.001 |
| **LDH, (U/L)** | 257.00 (182.00, 328.50) | 287.00 (233.00, 396.00) | 0.007 |
| **IgG, (g/L）** | 12.94 (9.95, 14.57) | 11.20 (9.82, 14.05) | 0.626 |
| **C3, (g/L)** | 0.99 (0.89, 1.24) | 0.87 (0.73, 1.11) | 0.059 |

**eTable 2.Comparison of medications used by the recurrent population and the non-recurrent population**

| **Variables** | **Total (n = 93)** | **Non-ruccrence**  **(n = 70)** | **Ruccrence**  **(n = 23)** | **Statistic** | ***P*** |
| --- | --- | --- | --- | --- | --- |
|
| **Prednisone (mg/d),Mean ± SD** | 57.66 ± 33.31 | 60.68 ± 33.48 | 48.48 ± 31.75 | t=1.54 | 0.13 |
| **Hydroxychloroquine,n(%)** |  |  |  | χ²=0.12 | 0.73 |
| **NO** | 77 (82.80) | 59 (84.29) | 18 (78.26) |  |  |
| **YES** | 16 (17.20) | 11 (15.71) | 5 (21.74) |  |  |
| **Thalidomide,n(%)** |  |  |  | χ²=0.04 | 0.83 |
| **NO** | 86 (92.47) | 64 (91.43) | 22 (95.65) |  |  |
| **YES** | 7 (7.53) | 6 (8.57) | 1 (4.35) |  |  |
| **Cyclophosphamide,n(%)** |  |  |  | χ²=0.41 | 0.52 |
| **NO** | 68 (73.12) | 50 (71.43) | 18 (78.26) |  |  |
| **YES** | 25 (26.88) | 20 (28.57) | 5 (21.74) |  |  |
| **Cyclosporine, n(%)** |  |  |  | χ²=0.00 | 0.98 |
| **NO** | 83 (89.25) | 63 (90.00) | 20 (86.96) |  |  |
| **YES** | 10 (10.75) | 7 (10.00) | 3 (13.04) |  |  |
| **Mycophenolate mofetil,n(%)** |  |  |  | χ²=0.08 | 0.78 |
| **NO** | 88 (94.62) | 67 (95.71) | 21 (91.30) |  |  |
| **YES** | 5 (5.38) | 3 (4.29) | 2 (8.70) |  |  |
| **Tacrolimus，n(%)** |  |  |  | χ²=0.05 | 0.82 |
| **NO** | 84 (90.32) | 64 (91.43) | 20 (86.96) |  |  |
| **YES** | 9 (9.68) | 6 (8.57) | 3 (13.04) |  |  |
| **Methotrexate ,n(%)** |  |  |  | - | 0.57 |
| **NO** | 90 (96.77) | 67 (95.71) | 23 (100.00) |  |  |
| **YES** | 3 (3.23) | 3 (4.29) | 0 (0.00) |  |  |
| **Tofacitinib,n(%)** |  |  |  | - | 1.00 |
| **NO** | 89 (95.70) | 67 (95.71) | 22 (95.65) |  |  |
| **YES** | 4 (4.30) | 3 (4.29) | 1 (4.35) |  |  |
| **Stable period hormone dosage(mg/d)** | 5.60 ± 1.97 | 5.69 ± 2.05 | 5.33 ± 1.74 | χ²=0.76 | 0.45 |
| **Hormone reduction rate(mg/d/month)** | 4.91± 2.40 | 4.99 ± 2.52 | 4.70 ± 2.08 | χ²=0.49 | 0.62 |
| **t: t-test, χ²: Chi-square test, -: Fisher exact** | | | | | |
| **SD: standard deviation** | | | | | |

**eTable 3.Sensitivity analysis subgroup results**

|  | **ROC** | **95%CI** | **C index** |
| --- | --- | --- | --- |
| **Change the treatment plan** |  | | |
| **1 year** | 0.77 | 0.46 - 1.08 | 0.82 (0.66 - 0.97) |
| **2 year** | 0.81 | 0.59 - 1.04 |
| **3 year** | 0.88 | 0.69 - 1.06 |
| **Short follow-up time** |  | | |
| **1 year** | 1.00 | 1.00 - 1.00 | 0.91 (0.82 - 0.99) |
| **2 year** | 0.91 | 0.75 - 1.08 |
| **3 year** | 0.92 | 0.78 - 1.05 |
| **Lack of lung function data** |  | | |
| **1 year** | 1.00 | 1.00 - 1.00 | 0.85 (0.72 - 0.98) |
| **2 year** | 0.92 | 0.78 - 1.05 |
| **3 year** | 0.92 | 0.80 - 1.04 |

**eFigure 1.Decision curve of the prediction model for the recurrence risk of patients with IIM-ILD**


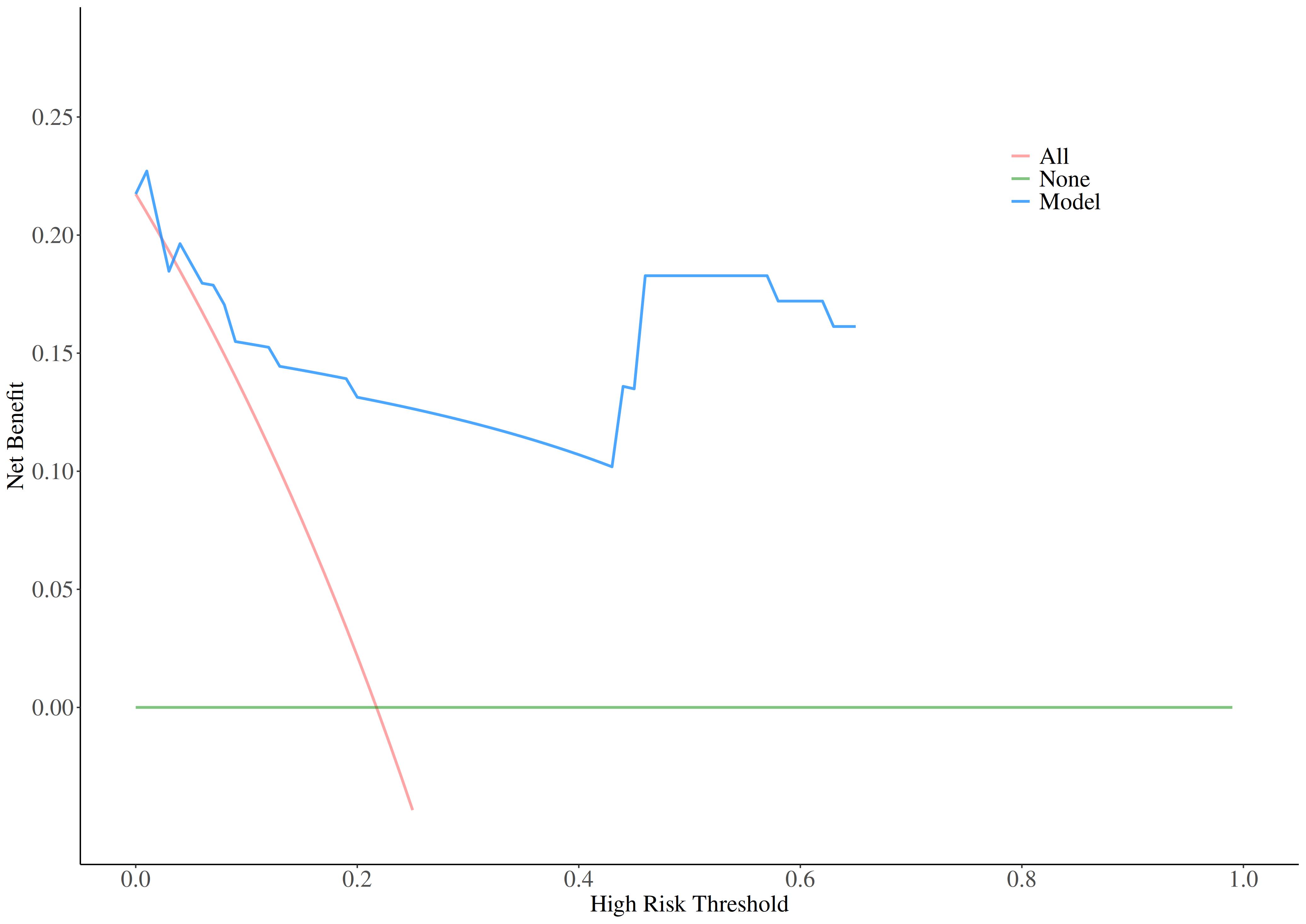


**1 year**


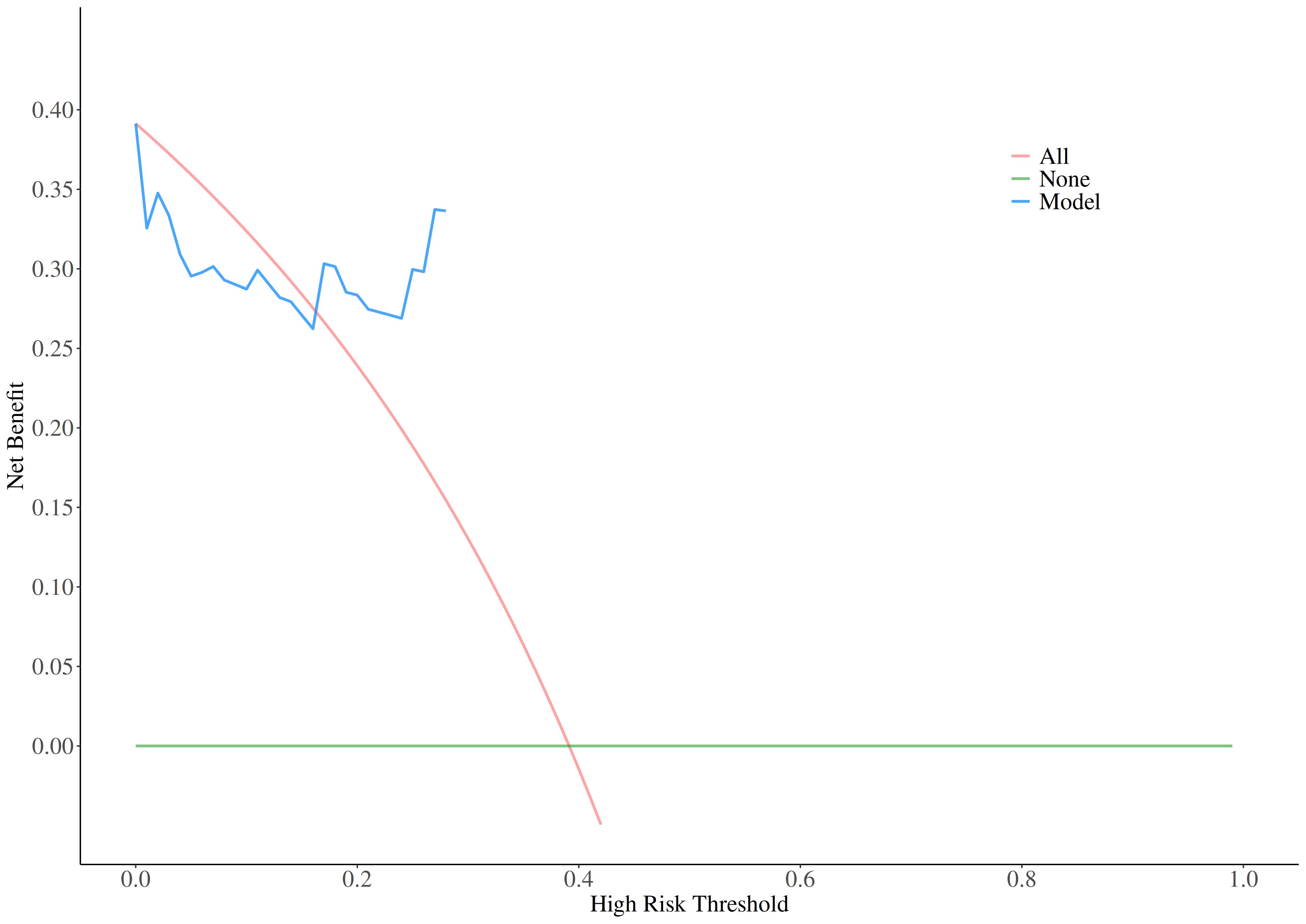


**2 years**


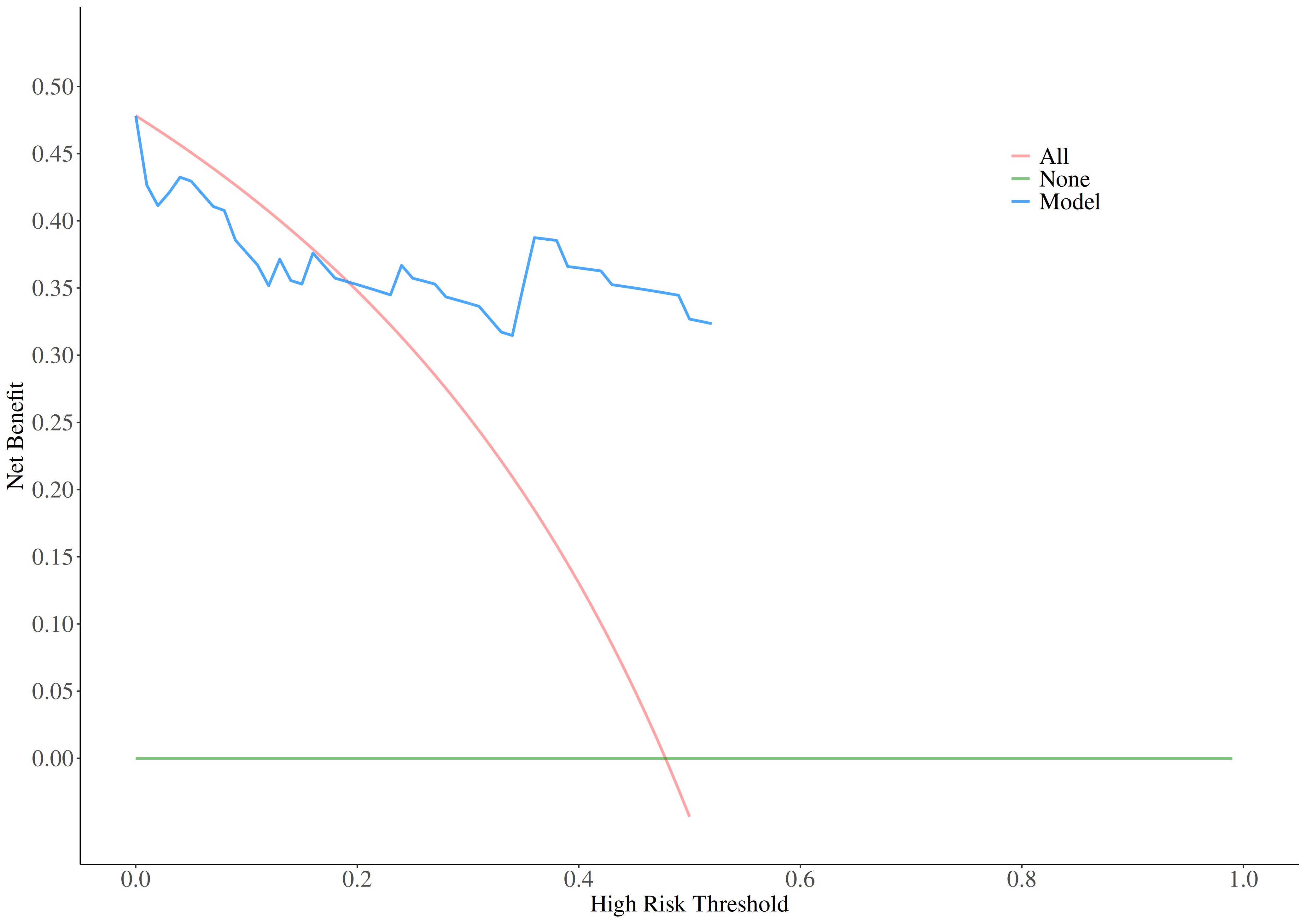


**3 years**

**eFigure 2.Calibration Curve of the Prediction Model for the Recurrence Risk of Patients with IIM-ILD(1,2,3 years)**


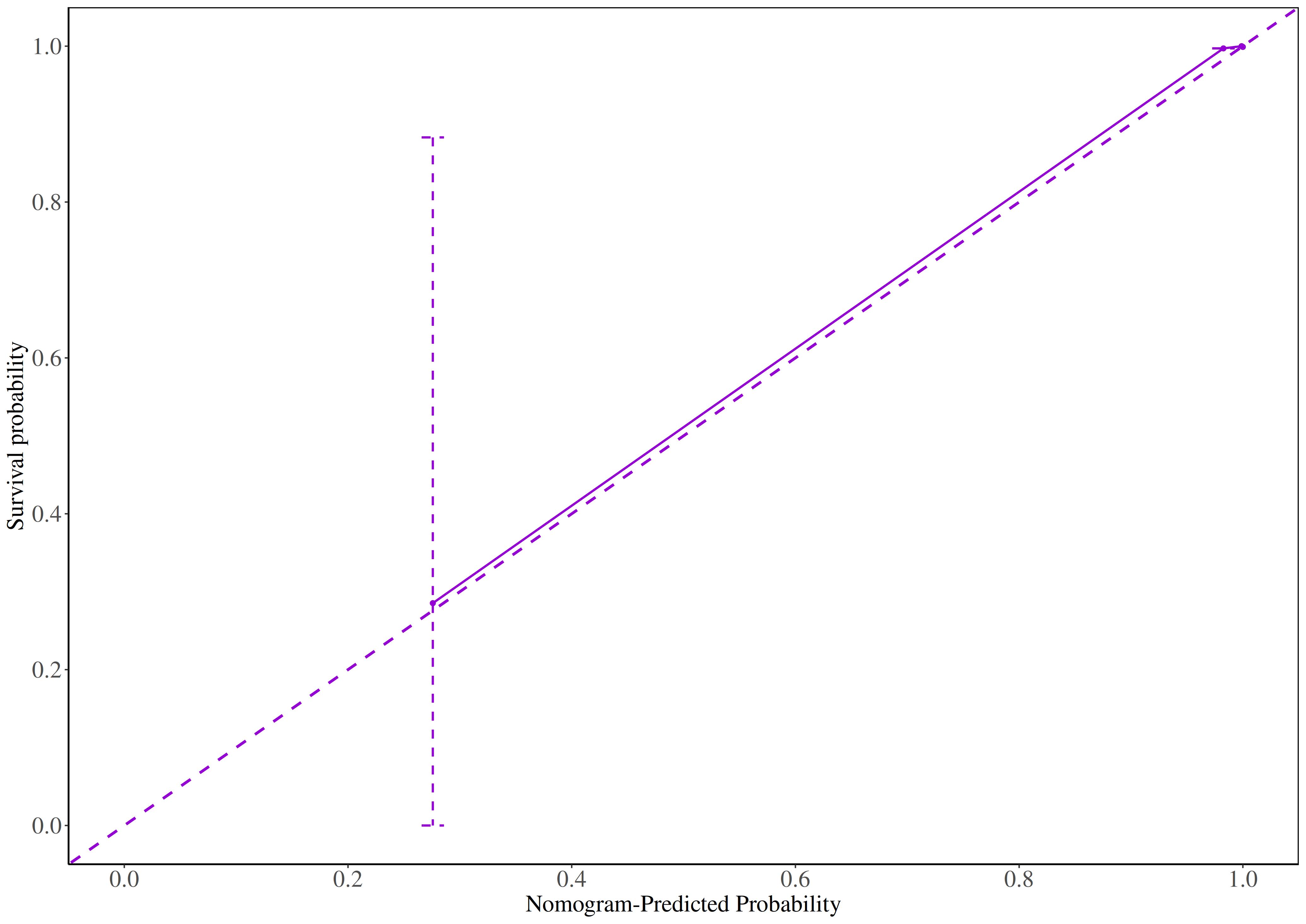


1 year


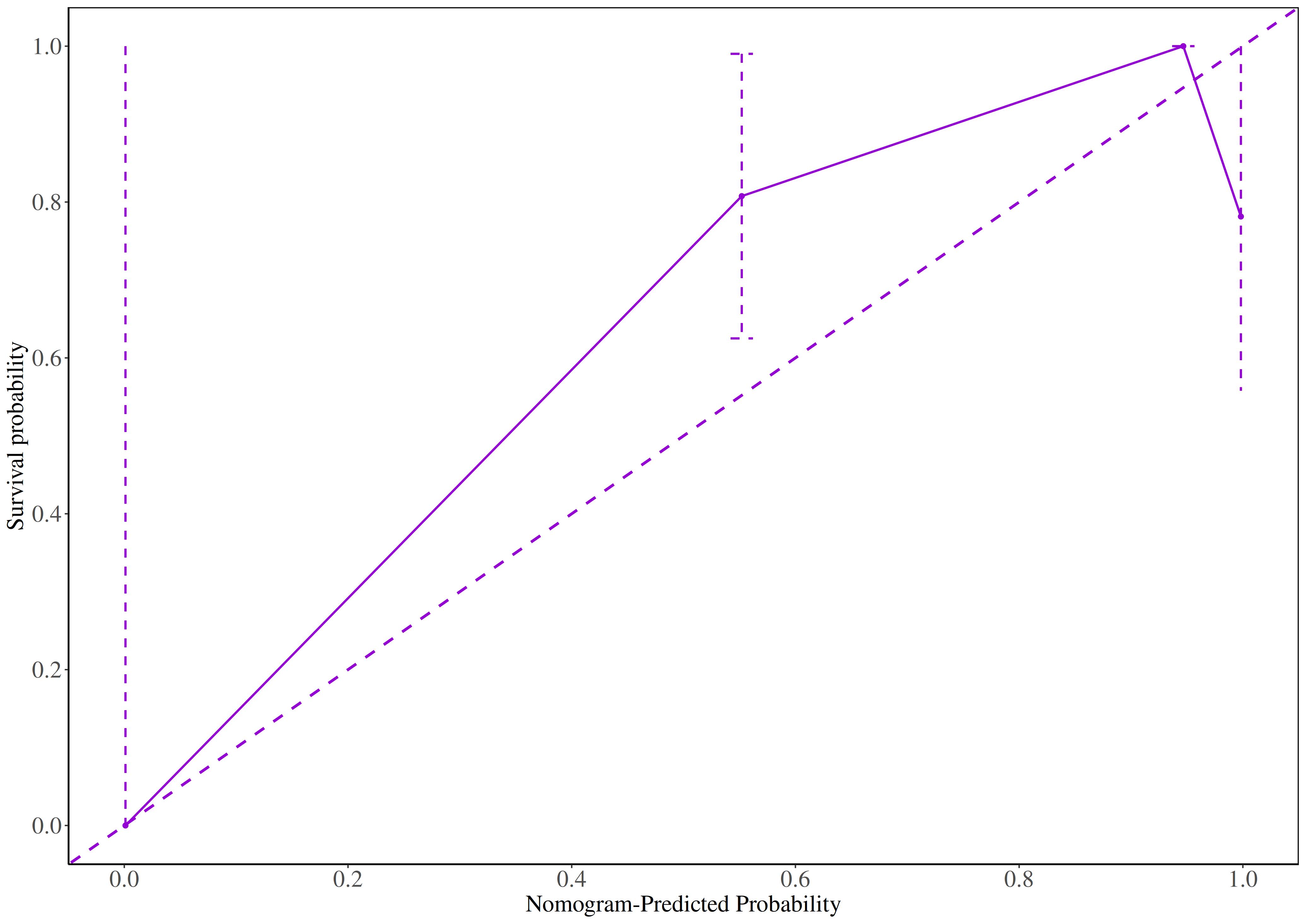


2 years


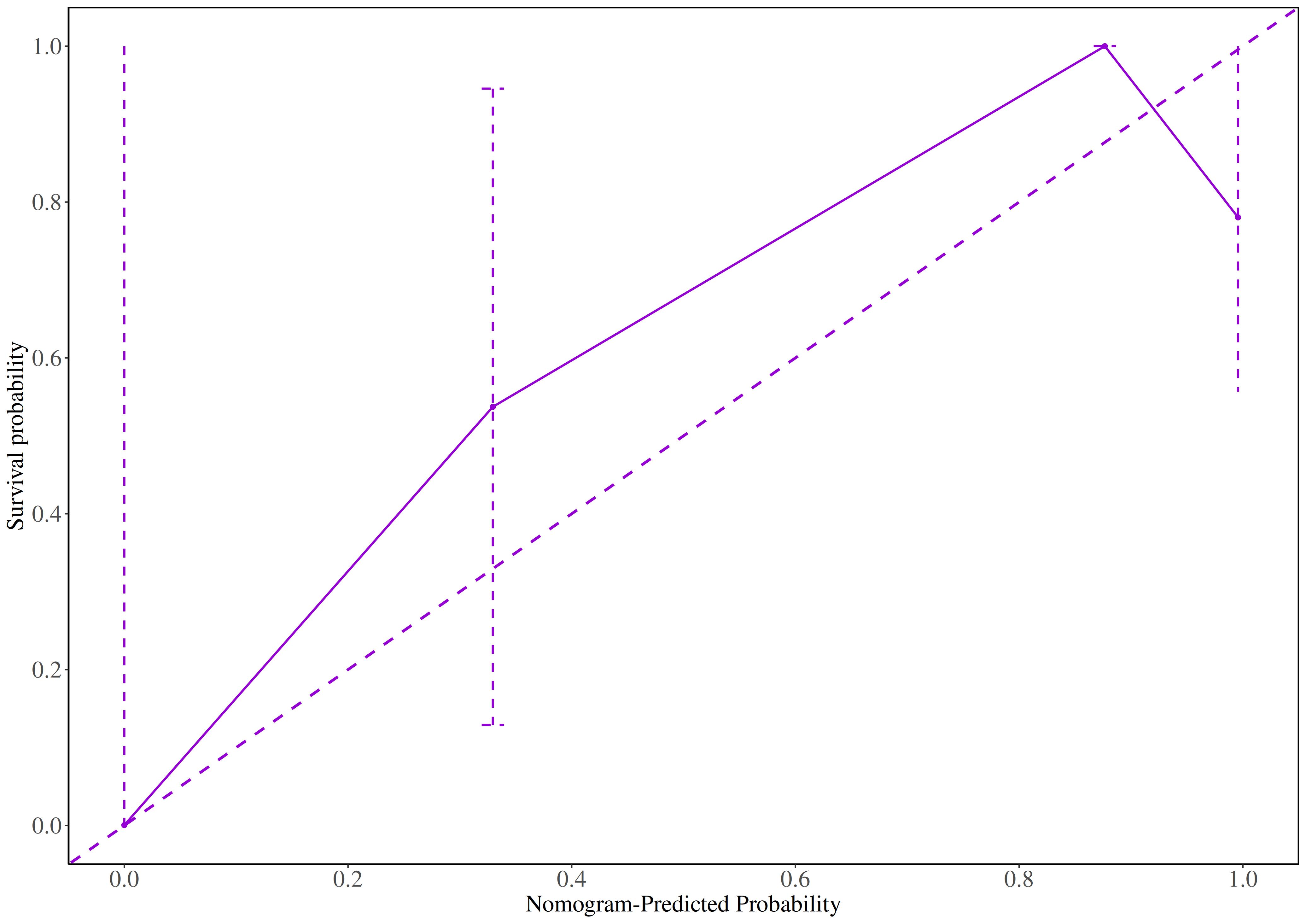


3 years

**eFigure 3.Kaplan-Meier (KM) Curve of the Recurrence Risk in Patients with IIM-ILD (Positive for RO-52, Positive for PL-7 and Risk Stratification)**


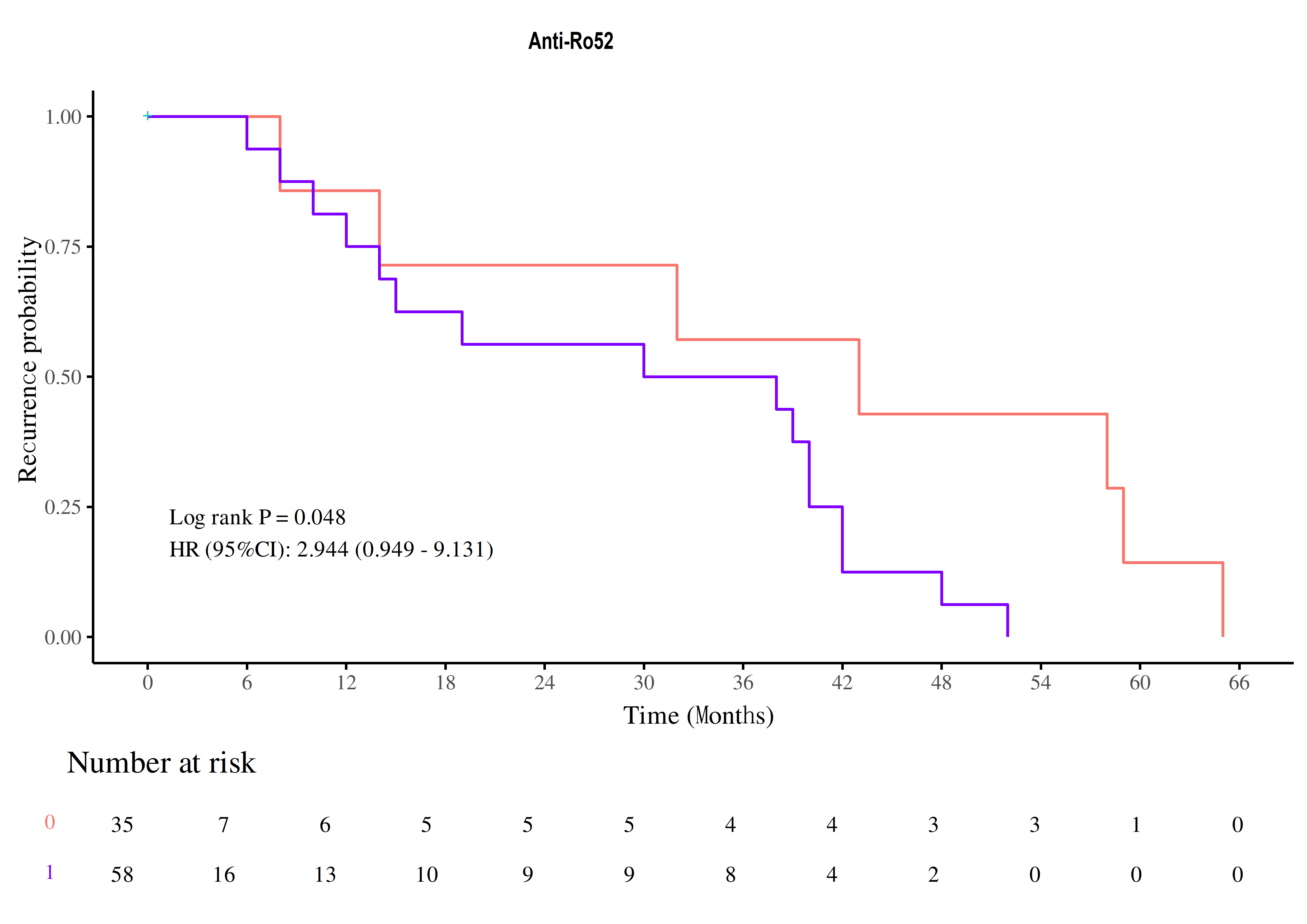

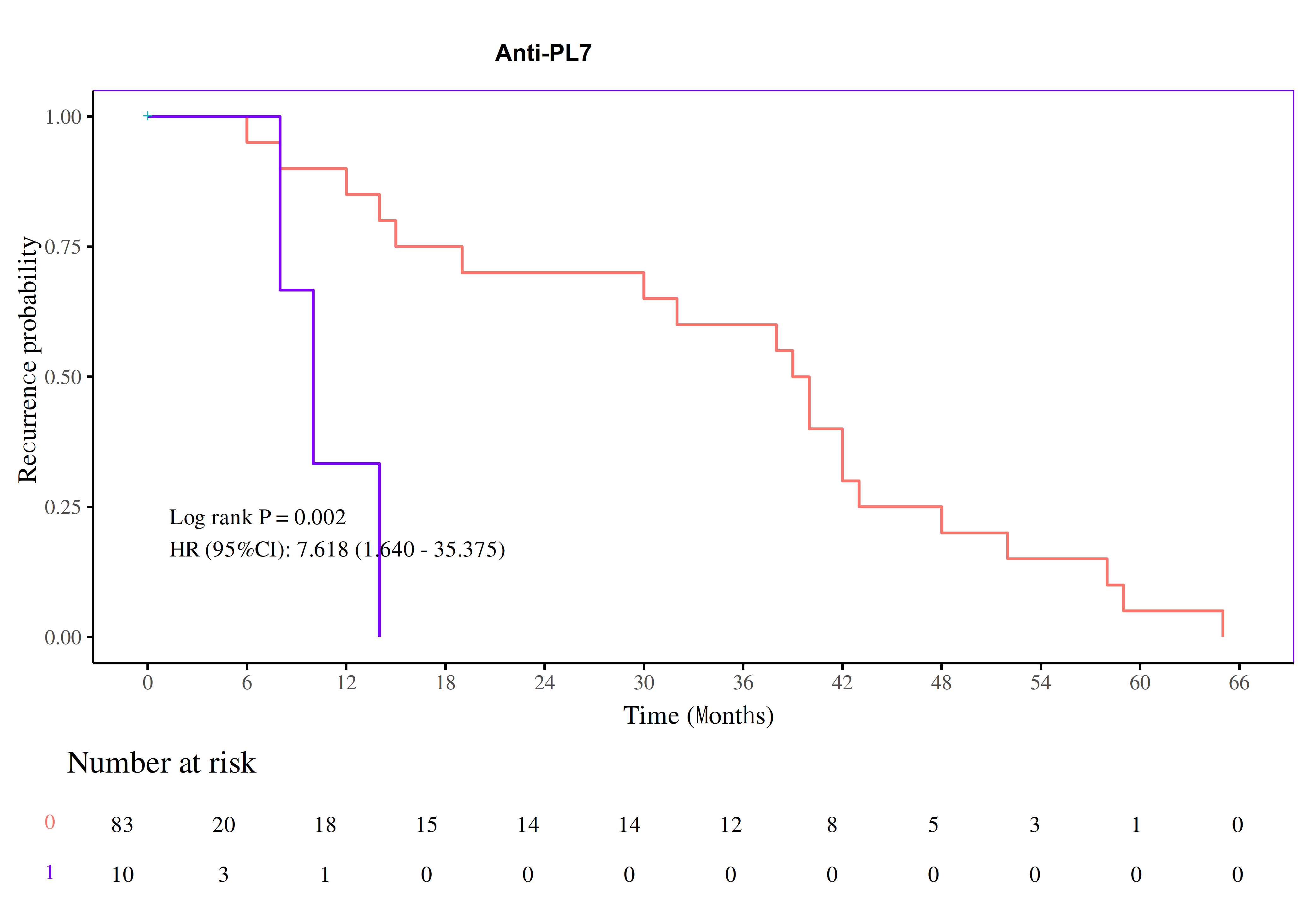

Supplement: Supplementary file 1 [file Table_1.DOC]
